# Multicellular rosettes link mesenchymal-epithelial transition to radial intercalation in the mouse axial mesoderm

**DOI:** 10.1016/j.devcel.2023.03.018

**Published:** 2023-04-19

**Authors:** Marissa L. Gredler, Jennifer A. Zallen

**Affiliations:** 1 Howard Hughes Medical Institute and Developmental Biology Program, Sloan Kettering Institute, New York, NY, USA; 2 Lead Contact

## Abstract

Mesenchymal-epithelial transitions are fundamental drivers of development and disease, but how these behaviors generate epithelial structure is not well understood. Here we show that mesenchymal-epithelial transitions promote epithelial organization in the mouse node and notochordal plate through the assembly and radial intercalation of three-dimensional rosettes. Axial mesoderm rosettes acquire junctional and apical polarity, develop a central lumen, and dynamically expand, coalesce, and radially intercalate into the surface epithelium, converting mesenchymal-epithelial transitions into higher-order tissue structure. In mouse *Par3* mutants, axial mesoderm rosettes establish central tight junction polarity but fail to form an expanded apical domain and lumen. These defects are associated with altered rosette dynamics, delayed radial intercalation, and formation of a small, fragmented surface epithelial structure. These results demonstrate that three-dimensional rosette behaviors translate mesenchymal-epithelial transitions into collective radial intercalation and epithelial formation, providing a strategy for building epithelial sheets from individual self-organizing units in the mammalian embryo.

## Graphical Abstract

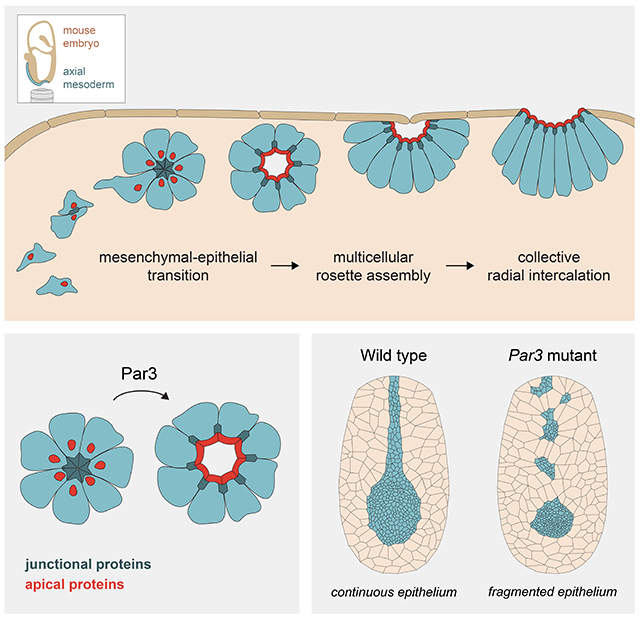

## Introduction

Epithelial tissues perform critical structural and barrier functions that are essential for the development of multicellular organisms^1,2^. The assembly of epithelial sheets is a fundamental event in embryonic development and drives tissue formation, growth, and remodeling through a variety of mechanisms. Cells build epithelial sheets through *de novo* polarization in the early embryo and can spontaneously form epithelial structures under certain conditions in culture^3,4^. In addition, the construction of functional epithelial organs often requires dynamic transitions between mesenchymal and epithelial cell states. Epithelial-mesenchymal transitions give rise to multiple cell lineages in the early embryo^5–9^, and the reciprocal mesenchymal-epithelial transitions are necessary to generate internal epithelial structures, promote organ growth and renewal, and when deregulated can contribute to tumor cell metastasis^10–14^. Although much is known about how epithelial identity is lost during epithelial-mesenchymal transition, it is not well understood how epithelial polarity, adhesion, and organization are established during mesenchymal-epithelial transition to drive the formation, expansion, and remodeling of epithelial tissues.

The genesis of an epithelial sheet requires the establishment of cell adhesion and apical-basal polarity, and in the case of radial intercalation, the integration of cells into an existing epithelial sheet^15,16^. Radial intercalation of individual cells promotes the incorporation of cells with specialized functions in the *Xenopus* mucociliary epithelium^17–23^, the mammalian airway^24^, and the *Drosophila* midgut^25,26^. In addition, the radial intercalation of many cells drives rapid phases of epithelial expansion in the vertebrate neural plate^27^, somites^28–31^, gut^32,33^, pancreas^34,35^, cochlea^36^, and kidney^37^. The insertion of single cells into epithelial sheets involves a wide range of cell behaviors, including directional cell migration^20^, microtubule and centriole repositioning^17,18,22^, actin-driven apical expansion^19,21^, filopodia-mediated stiffness sensing^23^, and the modulation of adhesion in both inserting cells and the target epithelium^25,26,32^. Adhesion between radially intercalating cells and the surrounding epithelium can be established at the apical surface^17,19^ or below the apical surface^25,26^ of the target epithelium in different examples of single-cell radial intercalation. However, it is not clear how large populations of cells integrate into epithelial structures without disrupting tissue integrity in systems where many cells undergo mesenchymal-epithelial transition and radial intercalation simultaneously.

Mesenchymal-epithelial transitions in the mouse axial mesoderm generate the node and notochordal plate, two epithelial structures with essential signaling functions^38–40^. The node, also known as the ventral node or posterior notochord^41^, is required to establish the left-right body axis of the mouse embryo through the directional rotation of node cilia^38,42^, and the notochordal plate patterns the dorsal-ventral axis of the brain and spinal cord by providing a localized source of the Sonic hedgehog morphogen^43^. Dynamic changes in the shape, location, and organization of axial mesoderm cells are necessary to generate these critical signaling structures. Axial mesoderm cells first undergo an epithelial-mesenchymal transition to delaminate from the epiblast epithelium^44–46^, followed by a mesenchymal-epithelial transition and radial intercalation into the endoderm epithelium at the distal surface of the embryo, generating the node and notochordal plate^47–50^. The notochordal plate then elongates along the anterior-posterior axis before internalizing to produce the rod-shaped notochord^47–49,51–54^. Although the lineage and differentiation of the axial mesoderm have been well-characterized^52,55,56^, how the axial mesoderm population undergoes mesenchymal-epithelial transition and radial intercalation to build a precisely positioned signaling center on the surface of the embryo is not understood.

Here we show that mesenchymal-epithelial transitions in the mouse axial mesoderm build an epithelial structure through the assembly and collective radial intercalation of multicellular rosettes. Cells in rosettes establish epithelial polarity through the sequential targeting of junctional and apical proteins to the central rosette domain, followed by the formation of an expanded internal lumen. Live imaging and three-dimensional image analyses show that rosettes dynamically form, expand, and coalesce below the embryo surface, producing elaborate cyst- and tunnel-like structures that integrate as units into the surface epithelium. In mouse embryos lacking the conserved polarity protein Par3 (Pard3), axial mesoderm cells form rosettes that acquire central tight junction polarity. However, rosettes fail to form an expanded apical domain and lumen, display altered dynamics and slowed radial intercalation, and ultimately form a small, fragmented node and notochordal plate epithelium. These results demonstrate that three-dimensional rosette assembly links mesenchymal-epithelial transition to collective radial intercalation to build a localized epithelial signaling center in the mouse embryo.

## Results

### Axial mesoderm cells display epithelial properties prior to radial intercalation

**Figure 1.**
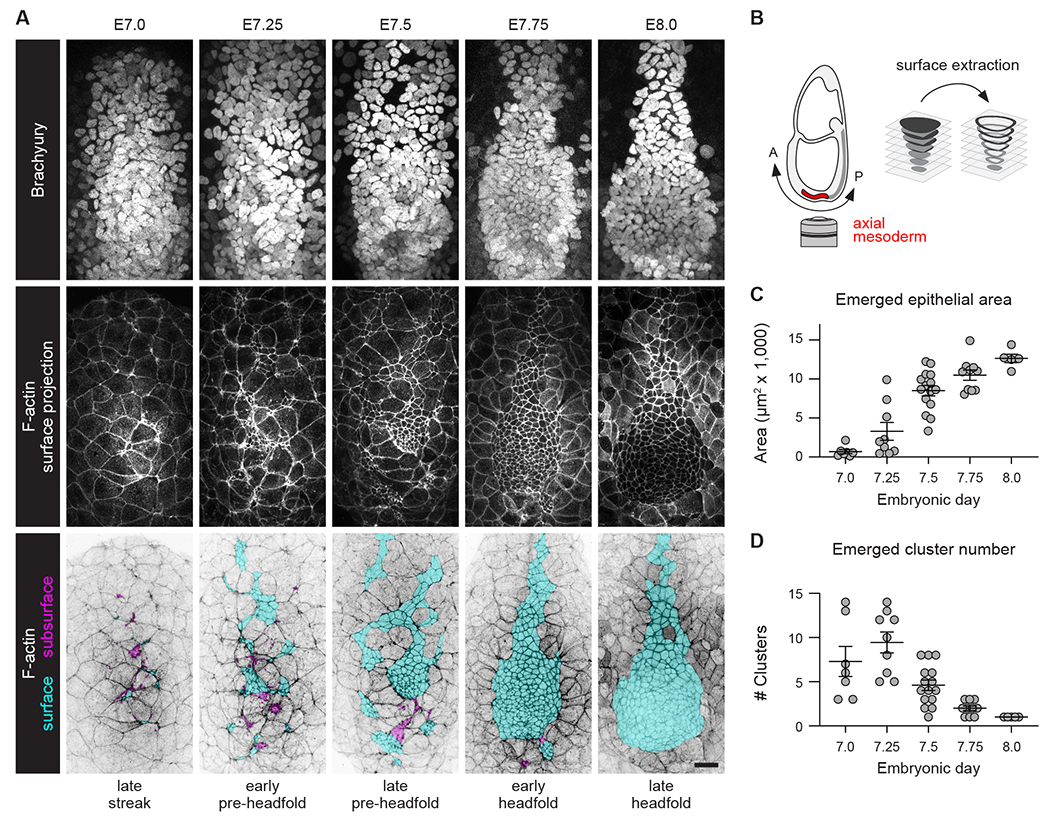
Emergence and coalescence of an epithelial sheet in the mouse axial mesoderm (A) Axial mesoderm cells in wild-type mouse embryos visualized with Brachyury (top row) and F-actin (phalloidin) (middle and bottom rows). Emerged axial mesoderm cells (cyan), unemerged axial mesoderm cells (magenta). Maximum-intensity projections (top and bottom rows). Surface projections (middle row). (B) Schematics of embryo orientation (left) and surface extraction method (right). Anterior (A), posterior (P). (C and D) Emerged apical area (C) and number of emerged clusters (D) in the axial mesoderm epithelium. Mean±SEM between embryos, each dot indicates one embryo (5-15 embryos/stage). Ventral views, anterior up. Bar, 25 μm. See also Figure S1 and Table S1.

To generate a surface epithelial structure, axial mesoderm cells need to establish adhesion with each other and with the surface endoderm, a hallmark of epithelial organization. To visualize the sites of epithelial assembly, we analyzed the localization of the adherens junction protein E-cadherin, the tight junction marker ZO-1-GFP^57^, and junction-associated filamentous actin (F-actin). To investigate whether axial mesoderm cells establish adhesion on or below the surface, we developed a computational method to distinguish the curved surface of the embryo from internal structures in three-dimensional confocal z-stacks, referred to as surface extraction (Figures 1A and 1B). Emerged axial mesoderm cells were identified by their smaller apical areas relative to the surface endoderm^49^ and their expression of the T-box transcription factor Brachyury^58^. The emerged axial mesoderm epithelium was first visible on the distal surface of the embryo at embryonic day (E) 7.0, where it displayed a relatively small apical surface area (688±257 μm^2^, mean±SEM) and was organized into several discrete clusters (7.3±1.7 clusters/embryo) (Figures 1C, 1D, and S1A–S1C; see Table S1 for a summary of all data). Over the next 24 hours, the apical area of the emerged axial mesoderm epithelium increased nearly 20-fold to occupy more than 10,000 μm^2^ on the embryo surface, and the axial mesoderm clusters coalesced to produce a single epithelial sheet (Figures 1C, 1D, and S1D). Thus, the axial mesoderm undergoes a dramatic reorganization over approximately one day of development to generate the node and notochordal plate.

**Figure 2.**
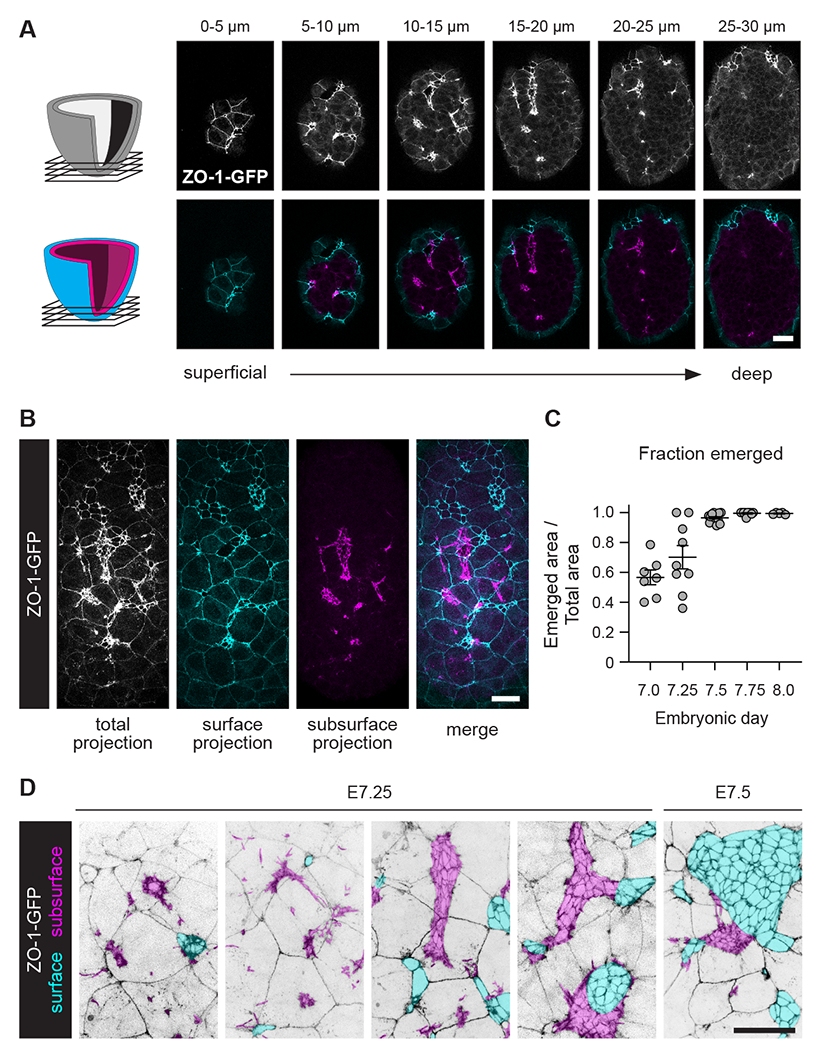
Axial mesoderm cells generate epithelial structures prior to emergence (A) ZO-1-GFP localization at different distances from the surface of an E7.25 embryo. Total signal (white), surface signal (cyan), and subsurface signal (magenta) (5 μm projections). (B) Maximum-intensity projection (white), surface projection (cyan), and subsurface projection (magenta) for the embryo in A. (C) Fraction of the axial mesoderm epithelium located on the embryo surface at the indicated stages. Mean±SEM between embryos, each dot indicates one embryo (5-15 embryos/stage). (D) ZO-1-GFP localization at different stages. Emerged cells (cyan), unemerged cells (magenta) (maximum-intensity projections). Ventral views, anterior up. Bars, 25 μm. See also Table S1.

Using the surface extraction method to distinguish between cell-cell junctions located on and below the embryo surface, we found that junctional structures detected with ZO-1-GFP, E-cadherin, and F-actin were present deep within the axial mesoderm population, before axial mesoderm cells appeared on the embryo surface (Figure 2A). Subsurface junctional structures displayed a wide range of morphologies, from discrete aggregates that were completely separate from the surface epithelium to expanded, sheet-like structures that were contiguous with the surface epithelial layer (Figures 2B–2D). Approximately one-third to one-half of the total apical area of the nascent axial mesoderm epithelium was positioned below the surface endoderm at E7.0 and E7.25, whereas the entire axial mesoderm epithelium was present on the surface at E8.0 (Figure 2C). These results show that axial mesoderm cells form junctions before they integrate with the surface endoderm, indicating that axial mesoderm cells establish adhesion prior to radial intercalation.

### Axial mesoderm cells transiently organize into cyst-like rosettes with central apical and junctional polarity

**Figure 3.**
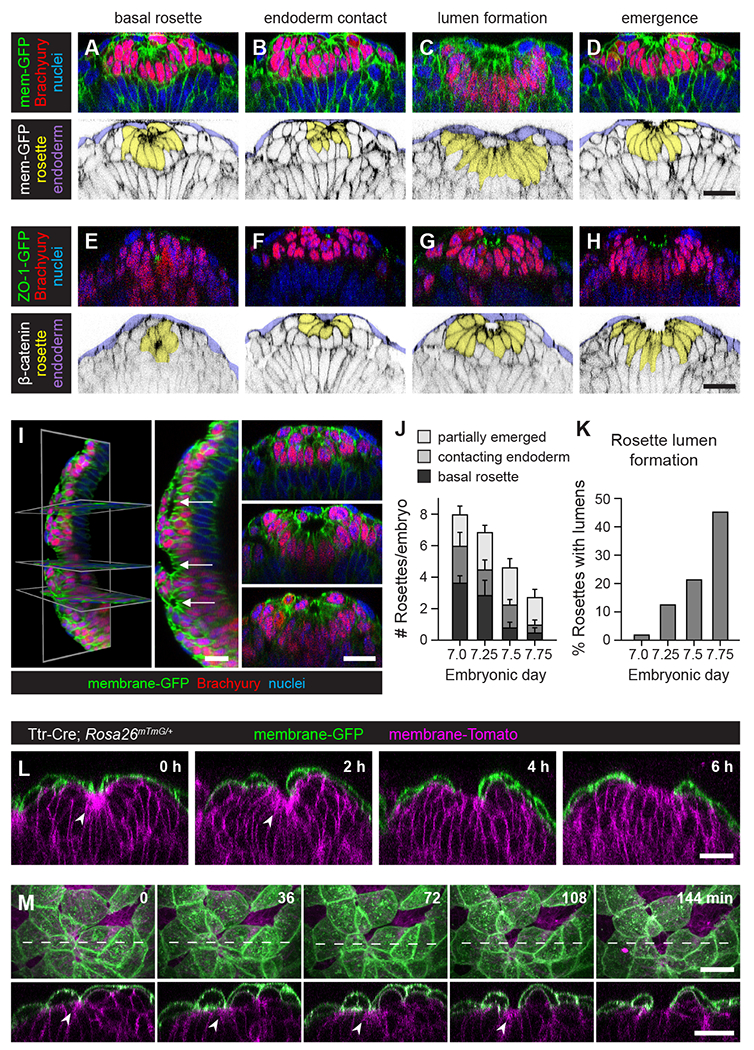
Axial mesoderm cells assemble into cyst-like rosettes that radially intercalate into the surface endoderm (A-D) Localization of membrane-GFP, Brachyury, and nuclei (Hoechst) (top panels). Axial mesoderm rosettes (yellow), surface endoderm cells (purple), membrane-GFP (black) (bottom panels). (E-H) Localization of ZO-1-GFP, Brachyury, and nuclei (Hoechst) (top panels). Axial mesoderm rosettes (yellow), surface endoderm cells (purple), β-catenin (black) (bottom panels). (I) Three-dimensional rendering of rosettes with internal lumens. Sagittal views, anterior up (left and middle panels), transverse views (right panels). Arrows indicate regions shown in right panels from top to bottom. (J) Number of basal, endoderm-contacting, and partially emerged rosettes/embryo at the indicated stages. Mean±SEM between embryos. (K) Percentage of rosettes with lumens at the indicated stages. (L and M) Time series of Ttr-Cre; *Rosa26*^*mTmG/+*^ embryos expressing membrane-GFP (green) in visceral endoderm cells and membrane-Tomato (magenta) in all other cells. Arrowheads indicate the rosette center; dotted lines indicate position of transverse views. Optically reconstructed transverse views in A-H, L, and M (bottom panels), Maximum-intensity projections, anterior up in M (top panels). 165 rosettes in 29 embryos in J and K. Bars, 25 μm. See also Figure S2, Video S1, and Table S1.

The finding that axial mesoderm cells establish adhesion before they merge with the surface endoderm suggests that critical events that initiate epithelial formation occur prior to radial intercalation. To investigate the mechanisms that promote epithelial formation in the mouse node and notochordal plate, we analyzed three-dimensional tissue organization using Brachyury to identify axial mesoderm cells and ZO-1-GFP or F-actin to mark cell junctions. Cell outlines were visualized with membrane-GFP in *Rosa26^mG/+^* embryos or by immunofluorescence for β-catenin. Axial mesoderm cells have been described to adopt conical or wedge shapes that assemble into fan-shaped groups or rosettes^48,50,52^. Consistent with these studies, we found that axial mesoderm cells formed rosette structures in which many cells were elongated toward a localized, membrane-rich domain (Figures 3A–3H). Distinct from published reports, rosettes were often detected well below the embryo surface. Classifying rosettes based on their proximity to the surface epithelium, we observed three types of rosette structures: basal rosettes were spherical in shape and consisted entirely of axial mesoderm cells (Figures 3A and 3E), endoderm-contacting rosettes contained at least one endoderm cell (Figures 3B and 3F) and often surrounded a central lumen (Figures 3C, 3G, and 3I), and partially emerged rosettes displayed a cup-shaped morphology and were contiguous with the surface epithelium (Figures 3D, 3H, and 3I). ZO-1-GFP and F-actin were enriched at the center of rosettes at all stages (Figures 3E–3H and S2A). These results indicate that axial mesoderm cells form multicellular rosettes in which the nascent cell junctions are oriented toward a common central domain.

To investigate whether rosette behaviors facilitate structural changes in the axial mesoderm, we first analyzed the timing of rosette formation. Rosettes were frequently observed at E7.0, before most axial mesoderm cells emerged on the embryo surface (8.0±1.2 rosettes/embryo) (Figure 3J). The number of rosettes decreased progressively over time (2.8±0.5 rosettes/embryo at E7.75) and rosettes were no longer detected after all axial mesoderm cells emerged on the embryo surface (10/10 embryos at E8.0). As the number of rosettes decreased, the percentage of rosettes with lumens increased, from less than 5% of rosettes at E7.0 to nearly half of the rosettes at E7.75 (Figure 3K). Smaller lumens were generally spherical in shape, whereas larger lumens formed tunnel-like structures that were often elongated along the anterior-posterior axis (Figures 3I, S2B, and S2C). These results indicate that axial mesoderm rosettes assemble and develop internal lumens prior to epithelial formation.

### Axial mesoderm rosettes are dynamic intermediates in radial intercalation

To determine whether rosette formation is a requisite intermediate in radial intercalation, we performed time-lapse imaging to visualize the behavior of axial mesoderm cells in live embryos. We used the *Rosa26^mTmG^* reporter^59^ and the Ttr-Cre driver^60^ to generate embryos that express membrane-GFP in the visceral endoderm and membrane-Tomato in all other cells, including the axial mesoderm. Intact, live embryos were dissected at E7.25, mounted distal side down in an inert collagen matrix, and imaged by confocal microscopy every 12 minutes for 8 hours in standard culture conditions^61,62^. Rosettes were identified as groups of membrane-Tomato-expressing axial mesoderm cells that were elongated toward a common membrane-rich domain. Individual rosettes were detected at varying stages of radial intercalation. A majority of rosettes observed radially intercalated into the surface endoderm during the imaging window (12/17 rosettes) (Figures 3L, 3M, S2D–S2F, and Video S1). Of the seven rosettes that were basal at the start of the movie, all became endoderm-contacting and four went on to fully emerge (Figures S2D and S2E). Of the five rosettes that were endoderm-contacting at the start of the movie, two partially emerged and three fully emerged during the movie (Figures 3M and S2F). Finally, five rosettes that started as partially emerged all fully emerged by the end of the movie (Figure 3L). Axial mesoderm cells that emerged as single cells were rarely observed, indicating that most radial intercalation events in the axial mesoderm occur through rosette intermediates. These results reveal that rosettes progress through a series of structural transitions over a several-hour timescale as they move toward and fuse with the surface epithelium.

The radial intercalation of axial mesoderm rosettes is predicted to disrupt the organization of the surface epithelium, raising the question of how axial mesoderm cells interact with the endoderm layer to transform from radially polarized cysts into a flat sheet. In particular, endoderm-contacting rosettes contain both mesoderm and endoderm cells and could facilitate the integration of axial mesoderm rosettes into the surface epithelium. To investigate the interactions between mesoderm and endoderm cells during radial intercalation, we took advantage of membrane-GFP expression in the visceral endoderm to visualize surface cell behavior. Before radial intercalation, endoderm cells were organized as a flat, squamous monolayer at the embryo surface. As rosettes approached the surface endoderm, a subset of endoderm cells became partially basally displaced and displayed a striking deformation toward the rosette center (16/17 rosettes tracked) (Figures 3L, 3M, S2D–S2F, and Video S1). The increased curvature of surface endoderm cells preceded the appearance of gaps in the surface epithelium, marking the site of rosette emergence (Figure 3M). As rosettes fused with the surface endoderm, the central rosette domain became contiguous with the apical domain of the surface epithelium, resulting in the dissolution of the radial rosette structure and conversion of the rosette into a flat sheet (Figures 3L, 3M, S2E, and S2F). Endoderm cells maintained extensive contacts with axial mesoderm cells throughout emergence, suggestive of strong attachment at the mesoderm/endoderm border. Together, these results demonstrate that axial mesoderm cells form multicellular rosettes that assemble under the surface endoderm, dynamically approach and interact with surface endoderm cells, and ultimately unfurl to produce a surface epithelial structure.

### *Par3* mutants form a discontinuous node and notochordal plate epithelium

**Figure 4.**
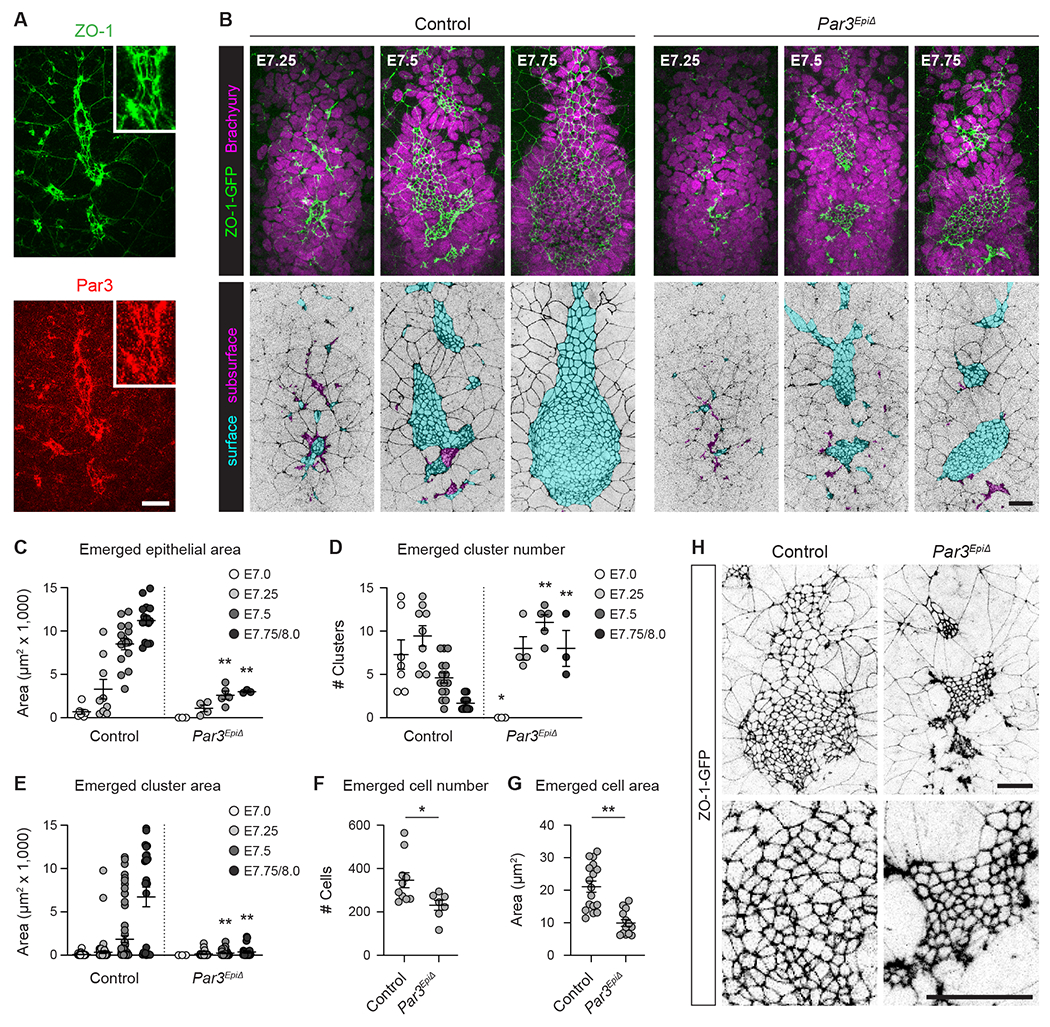
Par3 is required for epithelial organization in the mouse axial mesoderm (A) Localization of Par3 (red) and ZO-1 (green) in a control embryo at E7.25. (B) Localization of Brachyury (top panels) and ZO-1-GFP in control and *Par3*^*EpiΔ*^ embryos. Emerged axial mesoderm cells (cyan) and unemerged axial mesoderm cells (magenta). (C-E) Emerged epithelial area (C), emerged cluster number (D), and emerged cluster area (E) in control and *Par3*^*EpiΔ*^ embryos. Each dot indicates one embryo (C and D) or cluster (E). (F) Emerged cell number in control and *Par3*^*EpiΔ*^ embryos at E7.5. Each dot indicates one embryo. (G) Emerged cell area in control and *Par3*^*EpiΔ*^ embryos at E7.5. Each dot indicates the average cell area in one cluster. (H) Localization of ZO-1-GFP in control and *Par3*^*EpiΔ*^ embryos at E7.5. Ventral views, anterior up (maximum-intensity projections). Mean±SEM between embryos (C, D, and F) or clusters (E and G), *p<0.03, **p≤0.0003 compared to controls (unpaired t-test), 3-15 embryos/genotype at each stage. Controls in C-G show combined data for *Par3*^*EpiΔ*^ and *Par3*^*−/−*^ littermate controls. Bars, 25 μm. See also Figures S3–S5 and Table S1.

The transient organization of axial mesoderm cells into cyst-like rosettes suggests that rosette assembly could be a critical mediator of epithelial formation. To test this hypothesis, we examined the effects of removing a known regulator of cyst morphogenesis from the axial mesoderm. The PDZ-domain scaffolding protein Par3 is a conserved regulator of apical-basal polarity in epithelia and promotes lumen formation in several organisms, including the *C. elegans* spermatheca^63^, the mouse kidney^64^ and mammary gland^65^, the zebrafish neural tube^66,67^, the *Ciona* notochord^68^, and cultured epithelial cysts^69–71^. Par3 is required for the formation of epicardial progenitor cysts in the mouse heart^72^, and overexpression of Par3 can induce the formation of neuroepithelial rosettes in the chick neuroepithelium^73^. Par3 localizes to cell-cell junctions in axial mesoderm and surface endoderm cells during radial intercalation (Figure 4A). However, the functions of Par3 in the axial mesoderm and whether rosette-mediated morphogenesis and lumen formation are important for radial intercalation and epithelial formation are unknown.

To query the roles of Par3 in axial mesoderm morphogenesis, we used a *Par3* conditional allele (*Par3^flox^*)^74^ and the epiblast-specific Sox2-Cre driver^75^ to generate mouse embryos that lack *Par3* expression in all embryonic lineages (Sox2-Cre; *Par3^−/flox^*, referred to as *Par3^EpiΔ^* for epiblast-deleted) (Figures S3A and S3B). In addition, we used the Ttr-Cre driver to conditionally remove *Par3* from the visceral endoderm, the cell population that retains *Par3* expression in *Par3^EpiΔ^* mutants (Ttr-Cre; *Par3^flox/flox^*, referred to as *Par3^VEΔ^* for visceral endoderm-deleted). A single stripe of *Brachyury* expression was detected along the anterior-posterior axis of E9.5 *Par3^EpiΔ^* and *Par3^VEΔ^* embryos, indicating that the notochord is specified (Figure S3A). *Par3^VEΔ^* embryos were indistinguishable from controls, whereas *Par3^EpiΔ^* mutants had smaller heads, hypoplastic hearts, shorter body axes, and often failed to undergo axial rotation (Figures S3A and S3C), consistent with previous reports^72^. As we detected no anomalies in *Par3^VEΔ^* embryos, we continued our analyses with *Par3^EpiΔ^* mutants. Brachyury-positive cells were present in the distal region of the embryo in *Par3^EpiΔ^* mutants, similar to controls; however, the axial mesoderm population had irregular borders (Figures 4B and S3C–S3E). Despite these defects, axial mesoderm signaling activities in left-right and dorsal-ventral patterning remained functional in *Par3^EpiΔ^* mutants, as assessed by the left side-specific expression of *Pitx2* in the lateral plate mesoderm and induction of the floor plate marker Foxa2 in the ventral neural plate (Figures S4A and S4B). In addition, the notochordal plate later submerged to form the internal notochord in E8.5 *Par3^EpiΔ^* embryos, as in controls (Figure S4C). These data indicate that the node and notochord are specified and retain signaling competency in *Par3^EpiΔ^* mutants, but suggest defects in tissue organization.

To investigate the roles of Par3 in node and notochordal plate formation, we analyzed the organization of the axial mesoderm epithelium in *Par3^EpiΔ^* mutants. Axial mesoderm cells appeared on the distal surface of *Par3^EpiΔ^* embryos between embryonic days 7 and 8, similar to controls, and ZO-1-GFP localization provided evidence of junction formation (Figure 4B). However, the apical area of the emerged axial mesoderm epithelium was reduced by more than two-thirds in *Par3^EpiΔ^* mutant embryos at E7.5-E8.0 (Figure 4C), and emerged cells were organized into smaller, more numerous clusters (Figures 4D and 4E). Similar defects were observed in *Par3^−/−^* null mutants generated by germline excision of the *Par3* conditional allele (Figures S5A–S5G). To investigate the cellular basis of these defects, we analyzed the emergence and apical area of axial mesoderm cells. Fewer axial mesoderm cells were present on the surface of mutant embryos at E7.5 (Figure 4F), and a larger fraction of the epithelium remained unemerged at later stages, suggesting that radial intercalation is delayed in *Par3^EpiΔ^* mutants (Figures S5D and S5E). In addition, the average apical area of emerged cells was reduced by more than half in *Par3^EpiΔ^* mutants at E7.5 (9.9±1.0 μm^2^) compared with control cells (21.1±1.7 μm^2^) (Figures 4G and 4H). The total area of the axial mesoderm epithelium, including both emerged and unemerged regions, was reduced in *Par3* mutants (Figure S5B), consistent with the reduced apical area of individual cells. However, we cannot rule out the possibility that fewer cells undergo mesenchymal-epithelial transition in *Par3* mutants, due to the difficulty of counting cells with small apical domains. The axial mesoderm epithelium remained fragmented and did not recover to form a fully coalesced structure at later stages in *Par3^EpiΔ^* mutants (Figure S4C). These results indicate that axial mesoderm cells can establish cell adhesion and initiate epithelial organization in the absence of Par3 activity, but fail to assemble into a contiguous epithelial sheet.

### Par3 is required for epithelial dynamics and rapid radial intercalation in the axial mesoderm

**Figure 5.**
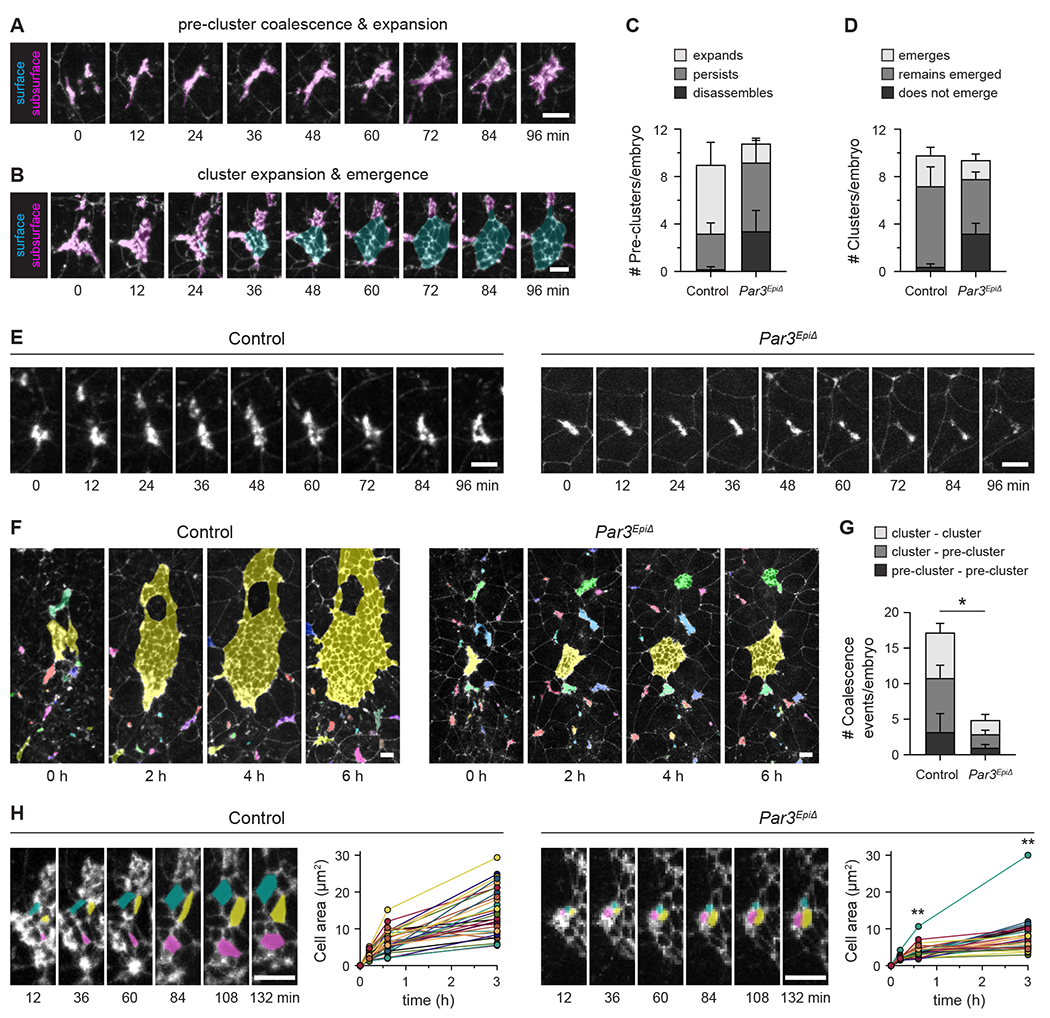
Live imaging reveals defects in epithelial expansion, coalescence, and emergence in *Par3* mutants (A and B) Stills from time-lapse movies of control embryos expressing ZO-1-GFP (white). Emerged axial mesoderm cells (cyan) and unemerged axial mesoderm cells (magenta). (C and D) Pre-cluster (C) and cluster (D) behaviors in control and *Par3*^*EpiΔ*^ embryos. (E) Time series of pre-cluster behaviors. (F) Time series of cluster and pre-cluster coalescence. Clusters and pre-clusters are highlighted in unique colors and adopt a shared color when two or more coalesce. (G) Number of coalescence events. (H) Time series and quantification of emerged apical cell area in control and *Par3*^*EpiΔ*^ embryos. The time point before the emerged apical surface was first visible was defined as t=0. Colors indicate three examples of cells that were first visible at t=12 min. Each line indicates one cell. Embryos were imaged starting at E7.25. Ventral views, anterior up (maximum-intensity projections). Mean±SEM between embryos, *p=0.03, **p≤0.0002 compared with controls (unpaired t-test), 3-5 embryos/genotype. Bars, 10 μm. See also Figure S6, Videos S2–S5, and Table S1.

The finding that axial mesoderm cells initiate epithelial formation but fail to generate a cohesive, expanded epithelial sheet in *Par3* mutants suggests that Par3 promotes critical behaviors necessary for epithelial organization. To investigate this possibility, we performed time-lapse imaging of control and *Par3^EpiΔ^* embryos expressing ZO-1-GFP (Videos S2–S5). Embryo morphology was not significantly disrupted by the live-imaging protocol, as verified by immunofluorescence at the end of the imaging session (Figures S6A and S6B). Two classes of epithelial structure, distinguishable by size and morphology, were evident in control embryos: small, dynamic ZO-1-GFP puncta referred to as pre-clusters and larger structures with clear epithelial morphology referred to as clusters (Figures 5A, 5B, Video S2, and Video S3). Comparison of the cellular organization in fixed embryos and time-lapse movies suggests that pre-clusters correspond to early rosettes and their precursors, whereas clusters correspond to later rosettes undergoing expansion and emergence (Figure S6C). No significant differences in the number of pre-clusters or clusters were observed between *Par3^EpiΔ^* mutants and controls (Figures 5C and 5D), indicating that epithelial formation is initiated in mutant embryos.

As *Par3^EpiΔ^* mutants initiate epithelial formation in the axial mesoderm but fail to form a continuous epithelial sheet, we reasoned that Par3 could mediate later steps in epithelial assembly, stability, or dynamics. To test whether Par3 is required to assemble epithelial precursors into a cohesive surface epithelial structure, we tracked pre-clusters and clusters in time-lapse movies. Tracking analysis revealed two defects in pre-cluster dynamics in *Par3^EpiΔ^* mutants. In control embryos, 64% of pre-clusters expanded to form clusters during the 7-hour imaging window, whereas only 15% of pre-clusters expanded in *Par3^EpiΔ^* mutants (Figure 5C). In addition, nearly one-third of pre-clusters disassembled or dissociated in mutant embryos, a behavior rarely observed in controls (Figures 5C and 5E). These defects are not the result of increased cell death, as no difference in the abundance of apoptotic cells was detected in *Par3^EpiΔ^* mutants compared to controls by cleaved caspase-3 staining (Figure S6D). These results indicate that Par3 is required for the expansion and stability of epithelial pre-clusters in the axial mesoderm.

To test whether Par3 is required for subsequent steps in epithelial formation, we analyzed the behaviors of epithelial clusters in *Par3^EpiΔ^* mutants. In control embryos, ZO-1-GFP clusters and pre-clusters frequently coalesced on and below the embryo surface (17.8±4.6 coalescence events/movie) (Figures 5F, 5G, and Video S4), producing elaborate structures reminiscent of epithelial cysts and tunnels in fixed embryos (Figures S6C and S6E). By contrast, the number of coalescence events was reduced by more than 70% in *Par3^EpiΔ^* mutants (Figures 5F, 5G, and Video S5). Epithelial clusters also displayed reduced or delayed emergence in *Par3^EpiΔ^* mutants. In control embryos, 87% of clusters that were initially below the surface (13/15 clusters) emerged during the 7-hour imaging window, whereas only 33% of subsurface clusters (8/24 clusters) emerged in mutant embryos (Figure 5D). In addition, emerged cells had significantly smaller apical areas in *Par3^EpiΔ^* mutants, with mutant cells reaching approximately half the apical area of control cells 3 hours after emergence, suggesting a defect in expansion or stability of the apical domain (Figure 5H). Together, these results demonstrate that Par3 is not required to initiate epithelial formation in the axial mesoderm; instead, Par3 is required for the expansion, coalescence, and timely emergence of axial mesoderm epithelial cells on the embryo surface.

### Par3 is required for aPKC localization and lumen formation in axial mesoderm rosettes

**Figure 6.**
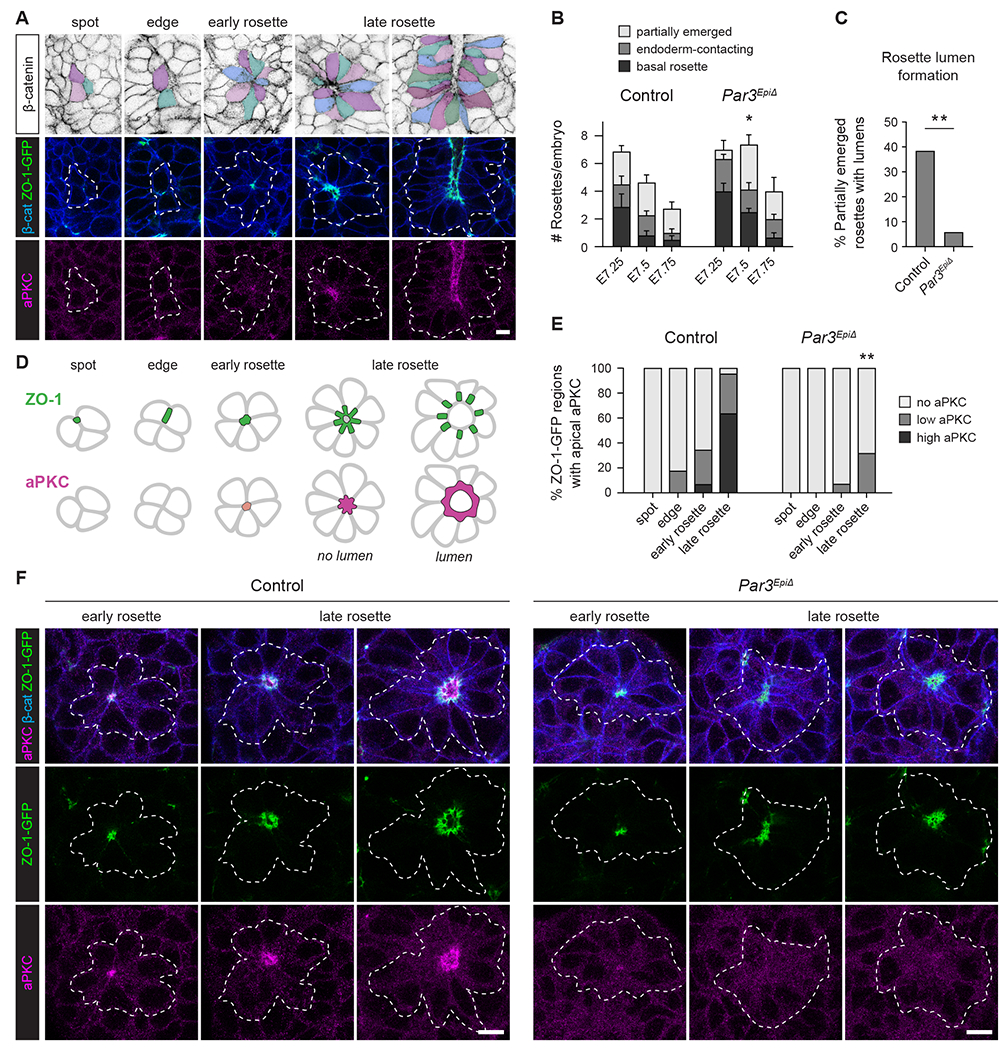
Par3 is required for apical aPKC localization and lumen formation in axial mesoderm rosettes (A) Localization of β-catenin, ZO-1-GFP, and aPKC during rosette formation. Pseudocolors (top panels) and dotted lines (middle and bottom panels) indicate cells in rosettes or cells flanking spots and edges. (B) Number of basal, endoderm-contacting, and partially emerged rosettes/embryo in control and *Par3*^*EpiΔ*^ embryos at the indicated stages. (C) Percentage of partially emerged rosettes with lumens in control and *Par3*^*EpiΔ*^ embryos. (D) Schematic of ZO-1 and aPKC localization in rosettes. (E) aPKC localization in the indicated ZO-1-GFP regions in control and *Par3*^*EpiΔ*^ embryos. (F) Localization of ZO-1-GFP, aPKC, and β-catenin in control and *Par3*^*EpiΔ*^ embryos. Dotted lines, rosette outlines. Ventral views, anterior up. Mean±SEM between embryos, *p=0.03 (unpaired t-test), **p≤0.0008 (Fisher’s exact test), 92-117 rosettes in 14-23 embryos/genotype in B, 34-52 rosettes in 14-23 embryos/genotype in C, and 58-92 ZO-1-GFP regions in 8 embryos/genotype in E. Control data in B are reproduced from Figure 3J. Bars, 10 μm. See also Figure S7 and Table S1.

As epithelial formation in the axial mesoderm involves the assembly of multicellular rosette intermediates, the failure to build a continuous epithelial sheet in *Par3* mutants could reflect essential roles for Par3 in regulating rosette formation, structure, or dynamics. To investigate these possibilities, we analyzed the number and morphology of rosettes in fixed *Par3^EpiΔ^* mutant embryos. Axial mesoderm rosettes were present in *Par3^EpiΔ^* mutants and were detected at a similar frequency to controls; however, significantly fewer rosettes displayed an expanded central lumen (6% of partially emerged rosettes in *Par3^EpiΔ^* mutants, compared with 38% of partially emerged rosettes in controls) (Figures 6A–6C and S7A). These results indicate that Par3 is not required for rosette assembly, but is necessary for the formation or expansion of the rosette lumen.

Lumen formation in cultured epithelial cysts involves the stepwise establishment and remodeling of cell adhesion and polarity in response to apical and basolateral inputs^76–78^. In particular, atypical protein kinase C (aPKC) promotes the formation of the apical cellular domain. To investigate the mechanisms by which Par3 regulates lumen formation, we defined the sequence of events that establish apical-basal polarity in the axial mesoderm, using immunofluorescence for aPKC to visualize the apical domain, Par3 to visualize cell junctions, and the localization of ZO-1 or ZO-1-GFP as a marker of rosette progression (Figures 6A and 6D). Before rosette assembly, ZO-1 localized to small puncta and short linear domains at the cell membrane, referred to as spots and edges, respectively. By contrast, ZO-1 was concentrated in a focused domain at the center of early rosettes. In late rosettes, ZO-1 displayed a discontinuous distribution at cell-cell contacts adjacent to the expanding rosette apical domain, resembling spokes on a wheel. Par3 colocalized with ZO-1 in a majority of spots and edges and in 100% of early and late rosettes (Figure S7B), indicating that Par3 localizes to cell-cell junctions at an early stage of rosette formation. By contrast, aPKC was largely absent from ZO-1 spots and edges, but colocalized with ZO-1 in the central domain of a subset of early rosettes (Figures 6E and 6F). In addition, aPKC was strongly enriched at the internal lumen-facing membrane in late rosettes, colocalizing with and often extending further into the apical domain than ZO-1. These results indicate that ZO-1 and Par3 localize to junctions at an early stage of rosette formation, whereas aPKC recruitment to the apical membrane defines a distinct step in rosette maturation.

The finding that *Par3* mutants display defects in lumen formation suggests that Par3 directs molecular events required for formation or expansion of the rosette apical domain. To determine whether Par3 is required for specific steps in rosette progression, we examined the localization of junctional and apical proteins in *Par3^EpiΔ^* mutants. ZO-1 localized to cell junctions in spots, edges, early rosettes, and late rosettes in *Par3^EpiΔ^* mutants, as in controls (Figure 6F). By contrast, aPKC was only weakly recruited to the apical domain of late rosettes in *Par3^EpiΔ^* mutants (Figures 6E and 6F). Whereas 95% of late rosettes in control embryos displayed apical aPKC localization, apical aPKC was only detected in 32% of late rosettes in mutant embryos and, when present, often accumulated to a reduced extent (Figures 6E and 6F). These results indicate that Par3 is not required to recruit ZO-1 to forming junctions in rosettes, but is necessary for aPKC localization to the central rosette domain, consistent with a role in promoting the formation or expansion of the apical domain and lumen.

### Par3 is required for Pals1 relocalization from a cytoplasmic compartment to the apical cellular domain

**Figure 7.**
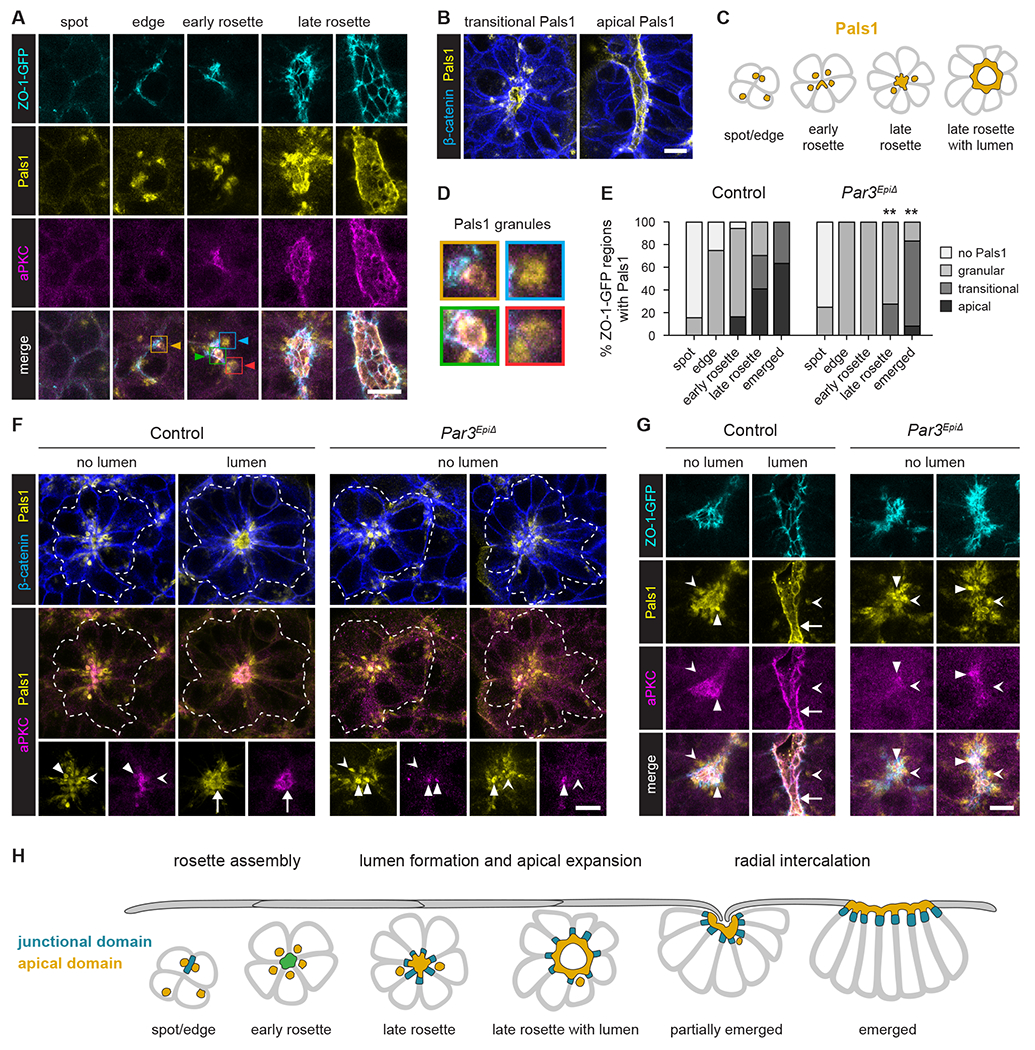
Par3 is required to relocalize Pals1 from a cytoplasmic compartment to the apical membrane (A) Localization of ZO-1-GFP, Pals1, and aPKC during rosette formation. Boxes and arrowheads indicate examples of Pals1 granules shown in D. (B) Localization of Pals1 and β-catenin in late rosettes. (C) Schematic of Pals1 localization in rosettes. (D) Close-ups of Pals1 granules in A. (E) Pals1 localization in the vicinity of the indicated ZO-1-GFP regions in control and *Par3*^*EpiΔ*^ embryos. No Pals1 detected (no Pals1), Pals1 in cytoplasmic granules (granular), Pals1 in granules and at the apical membrane (transitional), Pals1 primarily at the apical membrane (apical). (F) Localization of Pals1, β-catenin, and aPKC in control and *Par3*^*EpiΔ*^ embryos. Dotted lines, rosette outlines. (G) Localization of ZO-1-GFP, Pals1, and aPKC in control and *Par3*^*EpiΔ*^ embryos. White triangles, aPKC-positive Pals1 granules. Arrowheads, aPKC-negative Pals1 granules. Arrow, Pals1-positive apical membrane. (H) Model. Axial mesoderm cells form multicellular rosettes with central junctional and apical polarity that radially intercalate into the surface endoderm. Ventral views, anterior up (5 μm projections in A, B, and G, 5 μm projections for aPKC and Pals1 and single z-planes for β-catenin in F). **p=0.003 (Fisher’s exact test), 60-84 ZO-1-GFP regions in 6-7 embryos/genotype in E. Bars, 10 μm. See also Figure S7 and Table S1.

During lumenogenesis, apical determinants such as the transmembrane protein Crumbs and its cytoplasmic binding partners, Pals1 and Patj, are recruited to and promote the formation and expansion of the nascent apical domain^76–78^. To investigate the molecular mechanisms by which Par3 influences apical domain formation and lumen expansion in the axial mesoderm, we examined Pals1 localization during rosette assembly and emergence. Distinct from ZO-1, which exclusively localized to cell-cell junctions, Pals1 was detected at the apical membrane and in small (1-2 μm diameter) cytoplasmic granules that were generally located in close proximity to the membrane and were present at a frequency of approximately one per cell (Figures 7A, 7D, and S7C). Pals1 granules became clustered around the central domain in a majority of early rosettes, where they were concentrated in the apical cytoplasm surrounding the central ZO-1 junctions (Figures 7A–7E and S7C). During the early-to-late rosette transition, Pals1 localization shifted from cytoplasmic granules to the apical membrane (Figures 7A–7E). Late rosettes were detected at multiple stages of this transition, including rosettes with mostly granular, granular and apical (referred to as transitional), or mostly apical Pals1 distributions (Figures 7B and 7E). A subset of Pals1 granules was enriched for aPKC, including more than one-third of granules in late rosettes, indicating that these structures contain multiple apical proteins (Figures S7D–S7G). Pals1 granules were not detected in a majority of cells after emergence (Figure 7E), suggesting that these structures either disassemble or no longer contain Pals1 at later stages (Figures S7C, S7H, and S7I). These results demonstrate that Pals1 is recruited to the apical membrane of cells in late rosettes after transiently accumulating in an apical cytoplasmic compartment.

To determine if the recruitment of Pals1 to the apical membrane requires Par3 activity, we analyzed Pals1 localization in *Par3^EpiΔ^* mutants. Pals1 granules were readily detected in *Par3^EpiΔ^* embryos and were clustered around the central domain of early rosettes, as in controls (Figures 7E–7G). In addition, a subset of Pals1 granules in *Par3^EpiΔ^* mutants contained aPKC (Figure S7F and S7G). These results indicate that Pals1 granules are present and are targeted to the apical cytoplasm in *Par3* mutants. However, in contrast to controls, Pals1 granules often persisted in the cytoplasm adjacent to the rosette center and did not effectively relocalize to the apical membrane of late rosettes in *Par3^EpiΔ^* mutants (Figures 7E–7G). Whereas most late rosettes in control embryos had partially or exclusively apical Pals1 localization, Pals1 localization in late rosettes was primarily granular in mutant embryos (Figure 7E). In addition, Pals1 granules were often detected in emerged regions of mutant embryos, after granules were no longer present in a majority of emerged regions in controls (Figure 7E, S7H, and S7I). The finding that Pals1 granules localize to the apical cytoplasm in *Par3^EpiΔ^* mutants, but are not effectively delivered to the apical membrane, suggests that Par3 is required at a late step of rosette maturation to promote the expansion of the apical cellular domain.

## Discussion

Mesenchymal-epithelial transitions are essential drivers of epithelial formation and growth, but how these behaviors contribute to epithelial structure and remodeling is not well understood. Here we demonstrate that transient, lumen-containing rosettes represent intermediates in a dynamic series of behaviors that lead to epithelial formation in the mouse node and notochordal plate. Axial mesoderm rosettes dynamically assemble, expand, coalesce, and radially intercalate as units into the surface endoderm, producing a localized epithelial structure. We define the molecular events that establish epithelial polarity and show that axial mesoderm rosettes first develop a central tight junction domain, followed by expansion of a central apical domain and lumen. In *Par3* mutants, axial mesoderm rosettes assemble and establish tight junction polarity, but do not effectively recruit aPKC and Pals1 to the apical domain. Defects in apical domain expansion are associated with a disruption of rosette lumen formation and altered rosette dynamics in *Par3* mutants, including reduced coalescence and delayed radial intercalation, ultimately inhibiting the formation of a continuous epithelium. Together, these data demonstrate that three-dimensional rosette assembly translates mesenchymal-epithelial transitions into collective radial intercalation, providing a mechanism for generating epithelial structure from individual, self-organizing epithelial units in the mammalian embryo (Figure 7H).

Par3 intersects with a large number of cellular pathways involved in cell polarity, adhesion, and cytoskeletal organization that could mediate its effects on rosette dynamics and radial intercalation^79,80^. In particular, the apical polarity defects in *Par3* mutants suggest that apical expansion and lumen formation in three-dimensional rosettes represent critical steps that convert Par3-dependent cell polarization into dynamic structural changes that drive radial intercalation and epithelial formation in the mouse axial mesoderm. These defects are consistent with the well-established roles of Par3 in promoting the formation of the apical epithelial domain through direct interactions with aPKC^65,81–86^, indirect interactions with the Crumbs/Pals1/PatJ apical complex^87–89^, and by mediating exocyst-mediated membrane trafficking^90^. In one model, the apically directed transport of membrane, proteins, and fluids could generate expansive forces that drive rosette translocation to the embryo surface, like bubbles emerging at the surface of water. Alternatively, Par3 could influence rosette dynamics through effects on cell adhesion. Substantial junctional remodeling is predicted to be required for axial mesoderm rosettes to merge with the surface endoderm epithelium, reminiscent of the roles of Par3 in junctional organization and cell rearrangement in other systems^91–94^. In addition, epithelial pre-clusters often dissociate in *Par3* mutants, consistent with Par3 functions in establishing and stabilizing cell adhesion^95–98^. In a third model, Par3 could promote structural changes in the axial mesoderm by regulating cytoskeletal organization. For example, Par3 influences microtubule organization and centriole positioning^99–103^, which are essential for single-cell radial intercalation in *Xenopus*^18,22^. In addition, Par3 regulates contractile actomyosin networks that drive the formation and remodeling of planar rosettes in *Drosophila*^94,104^, chick^105^, zebrafish^106,107^, *Xenopus*^108^, mouse^108–110^, and *C. elegans*^111^. In the mouse embryo, mutants defective for the actin regulators Rac1 GTPase^112^ or the FERM-domain actin-binding protein Lulu/Epb4.1l5 ^50^ form a discontinuous node and notochordal plate, pointing to an important role for actin regulation in this process. Elucidation of the mechanisms that control rosette assembly and radial intercalation in the axial mesoderm will provide insight into the strategies that direct cell polarity and dynamic cell interactions during epithelial formation.

Lumen formation is essential for the structure and physiology of epithelial organs. Multiple cellular and molecular mechanisms of lumen formation have been described through *in vivo* studies and in cultured cells, including apoptosis^113,114^, oriented cell division^66,115–117^, and the dynamic accumulation of extracellular fluids^118–121^. Epithelial cells in culture have been shown to organize into radially polarized cysts through the recruitment and compartmentalization of conserved polarity complexes^76–78^. Disruption of Par3^69–71^, aPKC^116^, Par6B^116^, their upstream regulator Cdc42^114,115^, the Crumbs complex proteins Pals1^122^, Patj^123^, and Crumbs3^124,125^, or the membrane trafficking machinery^70,124^ produce aberrant cysts that form multiple lumens and often accumulate apical proteins in cytoplasmic vesicles, reminiscent of the defects in axial mesoderm cells in *Par3* mutants. In the mouse axial mesoderm, the apical proteins Pals1 and aPKC are delivered to the apical rosette domain after transiently accumulating in a cytoplasmic compartment. This compartment could correspond to recycling endosomes^70,125,126^, vacuolar apical compartments in unpolarized cells^69,127^, pre-assembled apical compartments that are continuous with the plasma membrane^25,26^, or another cytoplasmic structure. The finding that Par3 is required for apical expansion and lumen formation in the axial mesoderm highlights the role of apical expansion as a general strategy for lumen formation. The formation of a single lumen is essential for the development of several epithelial structures *in vivo*, including the mouse epiblast^128^ and zebrafish Kupffer’s vesicle, an organizer tissue that is functionally analogous to the mouse node^12,129–131^. In addition, the fusion of multiple lumens leads to the generation of tubular and branched epithelial structures^132^, including the zebrafish gut^119,133,134^ and neural tube^66,67,135^, the *Ciona* notochord^68,136^, and the mouse pancreas^34,35^, kidney^37^, and heart^72,137^. These results demonstrate that lumen formation is a basic unit of epithelial organization that can be mobilized to generate diverse epithelial structures.

During development, rosettes function as organizational intermediates that drive critical changes in tissue structure^104,138,139^. Here we demonstrate a role for transient, lumen-containing rosettes in driving radial intercalation events that translate mesenchymal-epithelial transitions into higher-order epithelial structure. In contrast to the radial intercalation of single cells, which are often broadly dispersed in the target epithelium, the radial intercalation of three-dimensional rosettes leads to the structural transformation of a pre-existing sheet by merging two fully formed epithelial structures. The collective intercalation of multicellular rosettes could accomplish a variety of functions to facilitate tissue remodeling. For example, rosettes could generate expansive forces that propel rosette movement and radial intercalation, support epithelial barrier function by allowing groups of cells to develop functional tight junctions before they join the surface epithelium, or promote cohesion or communication between axial mesoderm cells to generate a precisely positioned signaling structure within a larger epithelial sheet. Mesoderm and endoderm cells arise from different lineages, migrate along distinct trajectories, and are subject to different mechanisms of transcriptional and cytoskeletal regulation^140^, but in all cases their ability to form functional epithelial organs requires mesenchymal-epithelial transitions. It will be interesting to examine whether other mesenchymal cells form epithelial structures by individual or collective radial intercalation. Notably, similar cell behaviors drive epithelial formation in the chick lateral plate mesoderm, described in the accompanying study by Abboud Asleh and colleagues, in which mesenchymal-epithelial transition generates a wave of lumen-containing rosettes that fuse to produce opposing epithelial sheets in response to apical and basal polarizing cues^141^. Together with our results, these findings indicate that rosette assembly and remodeling represent a fundamental, self-organizing cell behavior, shared by multiple mesodermal lineages and functionally conserved across taxa, that could represent a universal mechanism for converting mesenchymal-epithelial transitions into large-scale epithelial organization.

### Limitations of the study

This study identifies three-dimensional rosettes as essential intermediates in epithelial formation in the mouse axial mesoderm through their roles in promoting collective radial intercalation. An independent study by Schultheiss and colleagues^141^ demonstrates a similar collective cell behavior in the chick lateral plate mesoderm, indicating that rosette assembly and remodeling represent a conserved strategy that converts mesenchymal-epithelial transitions into epithelial formation during embryonic development. However, several open questions remain. First, our findings identify the expansion of the apical cellular domain as a Par3-dependent process that could account for its roles in rosette dynamics and epithelial organization in the mouse axial mesoderm, although it is possible that additional roles of Par3 in cell adhesion, actomyosin contractility, or cytoskeletal organization contribute to the defects in radial intercalation and epithelial formation in *Par3* mutants. Second, the finding that the surface emergence of axial mesoderm cells is delayed but not inhibited in *Par3* mutants indicates that Par3 accelerates, but is not absolutely required for, radial intercalation. Thus, Par3-independent mechanisms also facilitate the radial intercalation of axial mesoderm rosettes, although these mechanisms promote slower emergence and result in the formation of a disjointed epithelial structure. Finally, our results do not rule out the possibility that earlier aspects of mesenchymal-epithelial transition could be affected in *Par3* mutants. In particular, the nature, location, and timing of the signals that induce mesenchymal-epithelial transition and rosette assembly in the axial mesoderm remain unknown. Mesenchymal-epithelial transition and rosette assembly may reflect intrinsic properties of axial mesoderm cells that are initiated prior to or during their migration through the primitive streak. Alternatively, mesenchymal-epithelial transition and rosette assembly could occur at a later stage in response to extrinsic signals provided by the overlying surface endoderm, the underlying neural plate, or adjacent mesoderm populations. Further studies of the mechanisms that govern mesenchymal-epithelial transition and rosette dynamics in the axial mesoderm will help to address these questions and shed light on how three-dimensional rosettes mediate epithelial organization during development.

## STAR METHODS

### RESOURCE AVAILABILITY

#### Lead contact

Further information and requests for resources and reagents should be directed to and will be fulfilled by the Lead Contact, Jennifer Zallen (zallenj@mskcc.org).

#### Materials availability

Materials generated in this study are available upon request.

#### Data and code availability

- All data reported in this paper will be shared by the lead contact upon request.
- No large-scale datasets or original code were generated in this study.
- Any additional information required to reanalyze the data reported in this paper is available from the lead contact upon request.

### EXPERIMENTAL MODEL AND SUBJECT DETAILS

#### Mouse strains and husbandry

The following mouse lines have been previously described: ZO-1-GFP (*Tjp1^tm1Tlch^*)^57^ (provided by Terry Lechler, Duke University), *Rosa26*^*mTmG* 59^, Ttr-Cre^60^ (provided by Kat Hadjantonakis, Sloan Kettering Institute), *Par3^flox^* (*Pard3^tm1c(KOMP)Wtsi^*) ^74^ (provided by Songhai Shi, Tsinghua University), and Sox2-Cre (*Edil3^Tg(Sox2-cre)1Amc^*)^75^. The *Rosa26^mG^* allele was generated by crossing Sox2-Cre females to *Rosa26^mTmG/mTmG^* males. The *Par3^flox^* allele contains loxP sites flanking exons 8 and 9; Cre-mediated recombination results in a frameshift mutation in downstream exons. The *Par3^−^* null allele (*Pard3^tm1d(KOMP)Wtsi^*) was generated by crossing Sox2-Cre females to *Par3^flox/flox^* males. The *Par3^flox^* and *Par3^−^* alleles were maintained on an FVB background. *Par3^EpiΔ^* embryos were generated by crossing Sox2-Cre; *Par3^−/+^* males to *Par3^flox/flox^* females to generate Sox2-Cre; *Par3^−/flox^* progeny. *Par3^EpiΔ^* mutant live-imaging experiments were performed using *Par3^flox/flox^; Tjp1^gfp/gfp^* females. Control embryos were stage-matched littermates with functionally wild-type (*Par3^+/+^*, *Par3^flox/+^*) or heterozygous (*Par3^Δ/+^*, *Par3^−/+^*) *Par3* genotypes. Embryonic day (E) 0.5 was defined as noon on the day of detection of a vaginal plug. Mice were bred and maintained under standard conditions in accordance with the Guide for the Care and Use of Laboratory Animals of the National Institutes of Health and approved by the Memorial Sloan Kettering Cancer Center Institutional Animal Care and Use Committee protocol 15-08-013.

### METHOD DETAILS

#### Embryo staging and genotyping

Embryo genotypes were determined by PCR using the indicated primers. Embryo stages were assigned based on the staging system published in Kaufman’s Atlas of Mouse Development Supplement^147^, as adapted from the Downs and Davies^148^ staging guide, with the exception that late streak (LS) and late streak, early allantoic bud (LSEB) stages were combined into a single late streak stage. For *Par3^EpiΔ^* and *Par3*^−/−^ embryos, early headfold and late headfold stages were combined into a single headfold stage based on the presence of visible headfolds and were compared to pooled early headfold and late headfold littermate controls. In the figures, embryo stages are labeled by approximate embryonic day as E7.0 (late streak), E7.25 (early pre-headfold), E7.5 (late pre-headfold), E7.75 (early headfold), and E8.0 (late headfold), although individual females produced embryos of varying stages.

#### Immunofluorescence

Whole mount immunofluorescence was performed on embryos dissected in ice-cold phosphate buffered saline (PBS) and fixed in either 4% paraformaldehyde (PFA) (Electron Microscopy Sciences) or 4:1 methanol (Thermo Fisher) and dimethylsulfoxide (DMSO, Sigma) at 4°C overnight. Primary antibodies were used to detect aPKC (Santa Cruz), β-catenin (BD Biosciences), Brachyury (Cell Signaling), cleaved caspase-3 (Cell Signaling), E-cadherin (Sigma), Foxa2 (Abcam), Par3 (clone 133 raised against amino acids 1-236 of mouse Par3, gift of James Fawcett, Dalhousie University)^142^, Pals1 (Proteintech), and ZO-1 (Developmental Studies Hybridoma Bank)^143^. ZO-1-GFP was visualized by residual fluorescence after fixation. After overnight fixation, methanol-fixed embryos were rehydrated in a graded methanol/PBS series (75%, 50%, 25%) for 30 min each before three 30-min washes in 0.1% Triton-X-100 (Thermo Fisher) in PBS (PBS-Tr), and PFA-fixed embryos were washed in five 1-h washes of 0.1% PBS-Tr. The following steps are shared by both fixation methods. After washing, embryos were incubated for 1 h in 5% bovine serum albumin (BSA, Sigma) and 10% goat serum (GS, Thermo Fisher) in PBS-Tr (5% BSA/10% GS/PBS-Tr) and then incubated overnight at 4°C in primary antibodies diluted in 5% BSA/10% GS/PBS-Tr. Primary antibodies were diluted 1:500, except antibodies to β-catenin (1:1,000), Par3 (1:400), and ZO-1 (1:100). The following day, embryos were washed with at least five 1-h washes of PBS-Tr, briefly equilibrated in 5% BSA/10% GS/PBS-Tr, and incubated in secondary antibodies diluted in 5% BSA/10% GS/PBS-Tr at 4°C overnight. AlexaFluor-conjugated secondaries (Invitrogen) were used at 1:1,000, AlexaFluor-546-conjugated phalloidin (Thermo Fisher) was used at 1:500, and Hoechst 33342 (1 mg/ml, Invitrogen) was used at 1:100. Following secondary antibody incubation, embryos were washed with at least five 1-h washes of PBS-Tr and stored at 4°C in PBS-Tr with 0.1% sodium azide before confocal imaging. Intact embryos were positioned in a 35 mm 1.5 glass-bottom dish (Cellvis) containing PBS and immobilized in triangular wells of pre-hardened 1% agarose; this temporary mounting method allowed for fine-scale repositioning to optimize embryo orientation during imaging.

#### *In situ* hybridization

Whole mount *in situ* hybridization was performed according to published methods^149^ and modified by replacing Triton X-100 with Tween-20 in KTBT solution and increasing the concentration of Triton X-100 in NTMT solution from 0.1 to 1%. The *brachyury* antisense riboprobe binds nucleotides 1,354-1,753 of *brachyury* mRNA (NM_009309_2) and was synthesized from a plasmid generously provided by Ruth Arkell (Australian National University). The *Pitx2* antisense riboprobe binds nucleotides 927-1,394 of *Pitx2* mRNA (NM_001042504.2).

#### Live embryo culture

Dissection steps for live embryo culture were performed in the following solutions warmed to 37°C. Prior to dissection, collagen matrices of 1 mg/ml collagen I (Thermo Fisher) in 1x Dulbecco’s modified eagle medium (DMEM; Thermo Fisher) with 25 mM NaOH (Sigma) were prepared in 35 mm glass-bottom dishes (1.5 coverglass, Cellvis), polymerized at 37°C for at least 30 min, washed three times with DMEM to remove unpolymerized collagen fibrils, and sterilized by UV irradiation in a tissue culture hood for 30 min. Fine forceps were used to pierce wells in the collagen matrix, and residual DMEM was replaced with rat serum (Envigo) for embryo culture. Uterine horns were removed from timed pregnant female mice and transferred to 10% rat serum in DMEM/F12+glutamax (Thermo Fisher). Individual decidua were halved longitudinally and each embryo was dissected as an intact egg cylinder and ectoplacental cone, transferred to DMEM/rat serum in a collagen matrix well, and mounted with the distal surface of the egg cylinder resting on the coverglass. The culture medium was covered with a thin layer of mineral oil (Sigma) to prevent evaporation. Embryos were cultured at 37°C, 5% CO_2_ in stage-top incubators affixed to laser scanning confocal microscopes (PECON on Zeiss LSM700, Tokai HIT on Leica SP8) for up to 8 h. Confocal z-stacks were acquired every 12 min. Embryos were genotyped from tissue biopsies collected after culture and fixed in 4% PFA for immunofluorescence to confirm normal morphology.

#### Confocal imaging

Confocal microscopy was performed using 20x or 40x oil immersion objectives on Zeiss LSM700 (20x/0.8 Plan-APOCHROMAT, 40x/1.3 Plan-NEOFLUAR objective) or Leica SP8 (20x/0.75 HC PL APO, 40x/1.3 HC PL APO objective) confocal microscopes. Fixed and live whole embryos were imaged in 70-200 μm confocal z-stacks and acquired either with 4 μm optical slices and 2 μm z-steps for 20x objectives, 2 μm slices with 1 μm z-steps for 40x objectives, or 1 μm slices with 0.5 or 0.35 μm z-steps for 40x objectives. Young embryos were imaged in single z-stacks covering a region 194 μm wide by 388 μm tall, and larger embryos were imaged in multiple stacks that were stitched together after acquisition using the “Pairwise Stitching” Fiji plugin^145^. Light micrographs were acquired on a Canon EOS T7i camera affixed to a Zeiss Stemi 508 stereo microscope.

#### Image processing

Surface extraction is a custom, semi-automated computational method used to visualize the embryo surface in confocal z-stacks of fixed and live mouse embryos. Surface extraction was performed using Fiji^144^ in four steps: mask generation, mask blurring, mask subtraction, and surface projection. Mask outlines corresponding to the outer surface of the embryo were manually drawn as regions of interest (ROIs) using the brush and freehand selection tools. Outlines were drawn at varying intervals in the image stack (every 2 to 5 slices near the curved surface of the embryo, every 10 to 40 slices in deeper regions with lower surface curvature, and every slice in difficult-to-segment regions). A provisional stack of black images was created with the same dimensions as the original stack, upon which the ROIs (representing the embryo outline) were drawn in a white fill using the “fill” function. White-filled shapes were eroded by 9-12 μm using the “erode” function to exclude surface tissues from the ROI, and interpolated using the “Interpolate ROIs” function. This provisional stack was Gaussian blurred to smooth the transition between slices and subtracted from the original z-stack using the “Image Calculator” function to generate the final stack of surface slices. Finally, a maximum intensity projection of the resulting stack of surface slices was used to generate a surface projection to visualize the apical surface of the tissue. Subsurface signal was detected using the same method except that the processed Gaussian blurred stack was inverted to white background images with black-filled ROIs (representing the embryo interior) and subtracted from the original z-stack. Optically reconstructed transverse and sagittal views were generated from confocal image stacks using the “Reslice Stack” function in Fiji, using voxel dimensions equal to those of the original z-stack.

### QUANTIFICATION AND STATISTICAL ANALYSIS

#### Image analysis

Emerged axial mesoderm cells were identified by their smaller apical surfaces compared to endoderm cells on surface-extracted projections of fixed and live embryos. Analyses of emerged and unemerged epithelial regions, as distinguished by surface extraction, were performed on ZO-1-GFP-expressing embryos or embryos stained with E-cadherin or phalloidin. Emerged, unemerged, and total epithelial area and emerged cluster number in the axial mesoderm were quantified by drawing manual outlines in Fiji around all emerged and unemerged regions of all clusters observed within a 160 μm (mediolateral width) x 260 μm (anterior-posterior height) field of view centered on the ventral midline. For cell area measurements in fixed embryos, a single value was obtained for each cluster by dividing the total area of the cluster by the number of cells in the cluster, excluding highly constricted cells along the cluster perimeter.

Rosette and lumen analyses were performed using optically reconstructed transverse slices of confocal z-stacks generated in Fiji. Enrichment of ZO-1-GFP or F-actin was used to identify the rosette center. Rosette locations were assigned based on proximity of the rosette center to the embryo surface. Basal rosettes had central apical domains that were located at least one axial mesoderm cell diameter away from the surface endoderm; although the basolateral surface of cells in basal rosettes sometimes contacted the surface endoderm, their apical surface did not. In endoderm-contacting rosettes, one or more surface endoderm cells visibly contacted the rosette center. In partially emerged rosettes, the central rosette domain was open to the external embryo surface. Partially emerged rosettes were distinguished from fully emerged regions (which were not included in this analysis) by their cup-shaped morphology, wherein cells were collectively oriented toward a concave depression in the embryo surface. For rosettes with complex morphologies, rosette type was assigned based on the region of the rosette closest to the embryo surface; i.e. a rosette with both basal and endoderm-contacting regions was classified as endoderm-contacting. Lumen presence was appointed if clear negative space was detected within the apical region, marked by ZO-1 or F-actin, in at least one transverse xz plane. For lumen surface area and volume measurements, lumens were manually outlined and analyzed in optically reconstructed transverse views using Fiji.

In time-lapse movies of embryos expressing ZO-1-GFP, maximum intensity projections of confocal z-stacks were generated and the axial mesoderm clusters were manually annotated for tracking and characterization using the “TrakEM2” Fiji plugin^146^. ZO-1-GFP regions that contained cells with clear expanded apical domains were defined as clusters, and pre-clusters were defined as solid ZO-1-GFP domains, regardless of overall size and shape. Clusters and pre-clusters were tracked as distinct regions throughout the movie, including after any coalescence events. The acquired z-stacks were sufficiently deep to span all tissues of the distal embryo through to the empty space of the amniotic cavity; as such, the disappearance of any ZO-1-GFP expression was not caused by movement out of the field of view. Pre-cluster expansion was defined as the transition from a solid to a discontinuous pattern of ZO-1-GFP localization, accompanied by the appearance of visible single-cell outlines. Pre-cluster persistence was assigned to regions of uninterrupted ZO-1-GFP localization that neither expanded nor disappeared throughout the imaging window. Pre-clusters that disappeared or fragmented into multiple spots after the start of the movie or their inception were characterized as disassembling. Emergence was classified according to whether a cluster or pre-cluster was positioned below the surface at both 0 and 7 h (does not emerge), positioned on the surface at both 0 h and 7 h (remains emerged), or positioned below the surface at 0 h (or at the time when it was first detected) and was located on the surface at 7 h (emerges). Regions with partial emergence were scored as emerging if they became increasingly emerged between the 0 and 7 h time points; for example, clusters that moved from unemerged to partially emerged were scored as emerging, whereas clusters that remained partially emerged throughout the imaging period were reported in the remains emerged category. Coalescence events were counted when two or more individual ZO-1-GFP domains merged to form a single region, and were scored according to the morphology (pre-cluster or cluster) of each region at the time of coalescence. Apical expansion of individual cells after emergence was quantified by manually outlining single cells at three time points in Fiji (12 min, 36 min, and 180 min), and t = 12 min was the first time point at which the apical domain of the cell was discernible on the embryo surface. Apical cell areas were slightly larger in fixed embryos than at t = 3 h after emergence in time-lapse movies, presumably because cells continue to apically expand for several hours after emergence.

The localization of Par3, aPKC, and Pals1 relative to ZO-1 enrichment in fixed embryos was analyzed in a 73 μm (mediolateral width) x 145 μm (anterior-posterior height) field of view centered on the ventral midline. Protein localization was assessed in a sequential series of ten 5-μm thick z-projections generated in Fiji from confocal z-stacks of the distal-most 50 μm of the embryo. ZO-1-GFP-expressing embryos were used for analysis of aPKC and Pals1 localization, and immunofluorescence for ZO-1 was used for analysis of Par3 localization. Four morphological categories were defined based on ZO-1 enrichment: small puncta and short linear domains of ZO-1 at the membrane were referred to as spots and edges, respectively. Early rosettes were defined as uninterrupted ZO-1 localization at a central vertex of five or more cells, and late rosettes were assigned to regions of discontinuous ZO-1 expression with a webbed, latticed, or reticular pattern at the central vertex of multiple cells. Spot, edge, early rosette, and late rosette regions were first identified based on ZO-1-GFP or ZO-1 localization for each embryo, and the localization of aPKC, Pals1, and Par3 to the ZO-1-positive region or the apical membrane, in addition to the presence of adjacent Pals1 granules, was assessed.

#### Statistics and figure preparation

Statistical analysis was performed using the unpaired t-test and Fisher’s exact test functions in GraphPad Prism, and figures were assembled using Adobe Illustrator, InDesign, and Photoshop.

## Supplementary Material

Video S1 **Movie S1. Radial intercalation of an axial mesoderm rosette.** Related to Figure 3. Time-lapse movie of a Ttr-Cre; *Rosa26^mTmG/+^* embryo expressing membrane-GFP (green) in visceral endoderm cells and membrane-Tomato (magenta) in all other cells. Top, maximum intensity projection. Bottom, transverse view of the region indicated by the dotted line. Images were acquired every 12 min.

Video S2 **Movie S2. Assembly and emergence of the axial mesoderm epithelium.** Related to Figure 5. Time-lapse movie of a control embryo expressing ZO-1-GFP (white). Right, unannotated movie. Left, surface endoderm cells are annotated in shades of blue. Images were acquired every 12 min.

Video S3 **Movie S3. Dynamic organization of the emerging axial mesoderm epithelium.** Related to Figure 5. Time-lapse movie of a control embryo expressing ZO-1-GFP (white). Right, unannotated movie. Left, surface endoderm cells (annotated in shades of dark blue), unemerged axial mesoderm cells (cyan), emerged axial mesoderm cells (yellow). Images were acquired every 12 min.

Video S4 **Movie S4. Coalescence of epithelial structures in a control embryo.** Related to Figure 5. Time-lapse movie of a control embryo expressing ZO-1-GFP (white). Right, unannotated movie. Left, axial mesoderm clusters are annotated in different colors and turn yellow after coalescing with the main cluster. Images were acquired every 12 min.

Video S5 **Movie S5. Coalescence of epithelial structures in a *Par3* mutant embryo.** Related to Figure 5. Time-lapse movie of a *Par3^EpiΔ^* mutant embryo expressing ZO-1-GFP (white). Right, unannotated movie. Left, axial mesoderm clusters are annotated in different colors and turn yellow after coalescing with the main cluster. Images were acquired every 12 min.

Supplement **Table S1. Summary of data and statistical analyses.** Related to Figures 1–7 and S1–S7.

## Figures and Tables

**Table:** KEY RESOURCES TABLE

REAGENT or RESOURCE	SOURCE	IDENTIFIER
**Antibodies**		
mouse IgG2a anti-PKC-zeta (H-1)	Santa Cruz (H-1)	Cat#sc-17781; AB_628148
mouse IgG1 anti-β-catenin, clone 14	BD Biosciences (14)	Cat#610154; AB_397554
rabbit anti-Brachyury (D2Z3J)	Cell Signaling	Cat#81694; AB_2799983
rabbit anti-cleaved caspase 3 (5A1E)	Cell Signaling	Cat#9664; AB_2891355
rat anti-Uvomorulin/E-cadherin, clone DECMA-1	Sigma	Cat#U3254; AB_477600
rabbit anti-MPP5 (Pals1)	Proteintech	Cat# 17710-1-AP; AB_2282012
rabbit anti-Par3, clone 133	Wells et al.^142^	N/A
rat anti-ZO-1	Stevenson et al.^143^; Developmental Studies Hybridoma Bank (R26.4C)	AB_2205518
goat anti-mouse IgG2a, Alexa Fluor 546	Thermo Fisher	Cat#A-21133; AB_2535772
goat anti-mouse IgG1, Alexa Fluor 647	Thermo Fisher	Cat#A-21240; AB_2535809
goat anti-rabbit IgG, Alexa Fluor 488	Thermo Fisher	Cat#A-11034; AB_2576217
goat anti-rabbit IgG, Alexa Fluor 546	Thermo Fisher	Cat#A-11035; AB_2534093
goat anti-rabbit IgG, Alexa Fluor 647	Thermo Fisher	Cat#A-21245; AB_2535813
goat anti-rat IgG, Alexa Fluor 546	Thermo Fisher	Cat#A-11081; AB_2534125
		
**Chemicals, peptides, and recombinant proteins**		
Alexa Fluor 546 phalloidin	Thermo Fisher	Cat#A22283; AB_2632953
Bovine serum albumin (BSA), 30%	Sigma	Cat#A7284
Cultrex rat collagen I	Thermo Fisher	Cat#344010001
Dimethyl sulfoxide (DMSO)	Sigma	Cat#D8418
10x Dulbecco’s modified eagle medium	Sigma	Cat#D2429
Dulbecco’s modified eagle medium (DMEM)/F-12 + Glutamax supplement	Thermo Fisher	Cat#10565018
Goat serum	Thermo Fisher	Cat#16210064
Hoechst 33342	Thermo Fisher	Cat#H3570
Methanol	Thermo Fisher	Cat#A412-4
Mineral oil	Sigma	Cat#M8410
Paraformaldehyde (PFA)	Electron Microscopy Sciences	Cat#15710
Rat serum, whole embryo culture	Envigo	Cat#B4520
Triton-X-100	Thermo Fisher	Cat#327371000
		
**Experimental models: Organisms/strains**		
*Mus musculus*: FVB/N	The Jackson Laboratory	MGI:3609372 Stock 001800
*Mus musculus*: *Pard3*^*tm1c(KOMP)Wtsi*^ (*Par3*^*flox*^)	Liu et al.^74^	MGI:6270373
*Mus musculus*: *Pard3*^*tm1d(KOMP)Wtsi*^ (*Par3*^*—*^)	This paper	N/A
*Mus musculus: Gt(ROSA)26Sor*^*tm4(ACTB-tdTomato,-EGFP)Luo*^ (*Rosa26*^*mTmG*^)	Muzumdar et al.^59^; The Jackson Laboratory	MGI:3716464 Stock 007676
*Mus musculus*: *Edil3*^*Tg(Sox2-cre)1Amc*^ (Sox2-Cre)	Hayashi et al.^75^; The Jackson Laboratory	MGI:3716464 Stock 008454
*Mus musculus*: Tg(Ttr-Cre)1Hadj (Ttr-Cre)	Kwon and Hadjantonakis^60^	MGI:3829595
*Mus musculus*: *Tjp1*^*tm1Tlch*^ (ZO-1-GFP)	Foote et al.^57^	MGI:5558017
		
**Oligonucleotides**		
Primer: Cre-tg F: GCG-GTC-TGG-CAG-TAA-AAA-CTA-TC	The Jackson Laboratory, protocol 22392	N/A
Primer: Cre-tg R: GTG-AAA-CAG-CAT-TGC-TGT-TGT-CAC-TT	The Jackson Laboratory, protocol 22392	N/A
Primer: Cre-IPC F: 5’ CTA-GGC-CAC-AGA-ATT-GAA-AGA-TCT	The Jackson Laboratory, protocol 22392	N/A
Primer: Cre-IPC R: 5’ GTA-GGT-GGA-AAT-TCT-AGC-ATC-ATC-C	The Jackson Laboratory, protocol 22392	N/A
Primer: Par3-wt F: GGC-TCC-CTG-TTT-GCA-GGA-TA	Liu et al.^74^	N/A
Primer: Par3-wt R: CTG-GAC-CTT-GGC-AGT-GTG-TC	Liu et al.^74^	N/A
Primer: Par3-delta F: GCA-GAT-CAC-TTA-GTT-AAG-GGA	This paper	N/A
Primer: Rosa26-wt F: CTC-TGC-TGC-CTC-CTG-GCT-TCT	Muzumdar et al.^59^	N/A
Primer: Rosa26-wt R: CGA-GGC-GGA-TCA-CAA-GCA-ATA	Muzumdar et al.^59^	N/A
Primer: Rosa26-mTmG R: TCA-ATG-GGC-GGG-GGT-CGT-T	Muzumdar et al.^59^	N/A
Primer: Tjp1-wt F: CTT-TCA-GAT-GAT-TGT-AGC-CAG-C	Foote et al.^57^	N/A
Primer: Tjp1-wt R: CCT-TCA-TCA-GTT-CCA-ACA-AAT-GC	Foote et al.^57^	N/A
Primer: Tjp1-gfp F: GCT-TTC-AGA-TGA-TTG-TAG-CC	Foote et al.^57^	N/A
Primer: Tjp1-gfp R: GAA-CTT-GTG-GCC-GTT-TAC-GTC-G	Foote et al.^57^	N/A
		
**Software and algorithms**		
Adobe InDesign	Adobe	SCR_021799
Adobe Illustrator	Adobe	SCR_010279
Adobe Photoshop	Adobe	SCR_014199
Fiji	Schindelin et al.^144^	SCR_002285; https://imagej.net/software/fiji/
GraphPad Prism 9	GraphPad Software	SCR_002798
Pairwise Stitching	Preibisch et al.^145^	SCR_016568; https://imagej.net/plugins/image-stitching
TrakEM2	Cardona et al.^146^	SCR_008954; http://www.ini.uzh.ch/~acardona/trakem2.html
		
**Other**		
35 mm glass bottom dish, #1.5 cover glass	Cellvis	Cat#D35-20-1.5-N
		

## References

[1] Rodriguez-Boulan E, and Macara IG (2014). Organization and execution of the epithelial polarity programme. Nat Rev Mol Cell Biol 15, 225–242. 10.1038/nrm3775. 24651541 PMC4211427

[2] Buckley CE, and St Johnston D (2022). Apical-basal polarity and the control of epithelial form and function. Nat Rev Mol Cell Biol. 10.1038/s41580-022-00465-y. 35440694

[3] O’Brien LE, Zegers MM, and Mostov KE (2002). Opinion: Building epithelial architecture: Insights from three-dimensional culture models. Nat Rev Mol Cell Biol 3, 531–537. 10.1038/nrm859. 12094219

[4] Sigurbjörnsdóttir S, Mathew R, and Leptin M (2014). Molecular mechanisms of de novo lumen formation. Nat Rev Mol Cell Biol 15, 665–676. 10.1038/nrm3871. 25186133

[5] Hay ED (2005). The mesenchymal cell, its role in the embryo, and the remarkable signaling mechanisms that create it. Dev Dyn 233, 706–720. 10.1002/dvdy.20345. 15937929

[6] Acloque H, Adams MS, Fishwick K, Bronner-Fraser M, and Nieto MA (2009). Epithelial-mesenchymal transitions: The importance of changing cell state in development and disease. J Clin Investig 119, 1438–1449. 10.1172/JCI38019. 19487820 PMC2689100

[7] Thiery JP, Acloque H, Huang RY, and Nieto MA (2009). Epithelial-mesenchymal transitions in development and disease. Cell 139, 871–890. 10.1016/j.cell.2009.11.007. 19945376

[8] Ferrer-Vaquer A, Viotti M, and Hadjantonakis AK (2010). Transitions between epithelial and mesenchymal states and the morphogenesis of the early mouse embryo. Cell Adh Migr 4, 447–457. 10.4161/cam.4.3.10771. 20200481 PMC2958623

[9] Francou A, and Anderson KV (2020). The epithelial-to-mesenchymal transition (EMT) in development and cancer. Annu Rev Cancer Biol 4, 197–220. 10.1146/annurev-cancerbio-030518-055425. 34113749 PMC8189433

[10] Gunasinghe NP, Wells A, Thompson EW, and Hugo HJ (2012). Mesenchymal-epithelial transition (MET) as a mechanism for metastatic colonisation in breast cancer. Cancer Metastasis Rev 31, 469–478. 10.1007/s10555-012-9377-5. 22729277

[11] Pei D, Shu X, Gassama-Diagne A, and Thiery JP (2019). Mesenchymal-epithelial transition in development and reprogramming. Nat Cell Biol 21, 44–53. 10.1038/s41556-018-0195-z. 30602762

[12] Amack JD (2021). Cellular dynamics of EMT: Lessons from live in vivo imaging of embryonic development. Cell Commun Signal 19, 79. 10.1186/s12964-021-00761-8. 34294089 PMC8296657

[13] Ng-Blichfeldt JP, and Roper K (2021). Mesenchymal-to-epithelial transitions in development and cancer. Methods Mol Biol 2179, 43–62. 10.1007/978-1-0716-0779-4_7. 32939713

[14] Thowfeequ S, Stower MJ, and Srinivas S (2022). Epithelial dynamics during early mouse development. Curr Opin Genet Dev 72, 110–117. 10.1016/j.gde.2021.11.006. 34929609 PMC7615355

[15] Keller RE (1980). The cellular basis of epiboly: An SEM study of deep-cell rearrangement during gastrulation in Xenopus laevis. J Embryol Exp Morphol 60, 201–234. 7310269

[16] Warga RM, and Kimmel CB (1990). Cell movements during epiboly and gastrulation in zebrafish. Development 108, 569–580. 10.1242/dev.108.4.569. 2387236

[17] Stubbs JL, Davidson L, Keller R, and Kintner C (2006). Radial intercalation of ciliated cells during Xenopus skin development. Development 133, 2507–2515. 10.1242/dev.02417. 16728476

[18] Werner ME, Mitchell JW, Putzbach W, Bacon E, Kim SK, and Mitchell BJ (2014). Radial intercalation is regulated by the Par complex and the microtubule-stabilizing protein CLAMP/Spef1. J Cell Biol 206, 367–376. 10.1083/jcb.201312045. 25070955 PMC4121976

[19] Sedzinski J, Hannezo E, Tu F, Biro M, and Wallingford JB (2016). Emergence of an apical epithelial cell surface in vivo. Dev Cell 36, 24–35. 10.1016/j.devcel.2015.12.013. 26766441 PMC4735878

[20] Szabo A, Cobo I, Omara S, McLachlan S, Keller R, and Mayor R (2016). The molecular basis of radial intercalation during tissue spreading in early development. Dev Cell 37, 213–225. 10.1016/j.devcel.2016.04.008. 27165554 PMC4865533

[21] Sedzinski J, Hannezo E, Tu F, Biro M, and Wallingford JB (2017). RhoA regulates actin network dynamics during apical surface emergence in multiciliated epithelial cells. J Cell Sci 130, 420–428. 10.1242/jcs.194704. 28089989 PMC5278671

[22] Collins C, Majekodunmi A, and Mitchell B (2020). Centriole number and the accumulation of microtubules modulate the timing of apical insertion during radial intercalation. Curr Biol 30, 1958–1964 e1953. 10.1016/j.cub.2020.03.013. 32243862 PMC7239760

[23] Ventura G, Amiri A, Thiagarajan R, Tolonen M, Doostmohammadi A, and Sedzinski J (2022). Multiciliated cells use filopodia to probe tissue mechanics during epithelial integration in vivo. Nat Commun 13, 6423. 10.1038/s41467-022-34165-0. 36307428 PMC9616887

[24] Rock JR, Onaitis MW, Rawlins EL, Lu Y, Clark CP, Xue Y, Randell SH, and Hogan BL (2009). Basal cells as stem cells of the mouse trachea and human airway epithelium. Proc Natl Acad Sci U S A 106, 12771–12775. 10.1073/pnas.0906850106. 19625615 PMC2714281

[25] Chen J, and St Johnston D (2022). De novo apical domain formation inside the Drosophila adult midgut epithelium. eLife 11, e76366. 10.7554/eLife.76366. 36169289 PMC9545526

[26] Galenza A, Moreno-Roman P, Su YH, Acosta-Alvarez L, Debec A, Guichet A, Knapp JM, Kizilyaprak C, Humbel BM, Kolotuev I, and O’Brien LE (2023). Basal stem cell progeny establish their apical surface in a junctional niche during turnover of an adult barrier epithelium. Nat Cell Biol 10.1038/s41556-023-0116-w. PMC10317055 36997641

[27] Davidson LA, and Keller RE (1999). Neural tube closure in Xenopus laevis involves medial migration, directed protrusive activity, cell intercalation and convergent extension. Development 126, 4547–4556. 10.1242/dev.126.20.4547. 10498689

[28] Pourquié O (2001). Vertebrate somitogenesis. Annu Rev Cell Dev Biol 17, 311–350. 10.1146/annurev.cellbio.17.1.311. 11687492

[29] Nakaya Y, Kuroda S, Katagiri YT, Kaibuchi K, and Takahashi Y (2004). Mesenchymal-epithelial transition during somitic segmentation is regulated by differential roles of Cdc42 and Rac1. Dev Cell 7, 425–438. 10.1016/j.devcel.2004.08.003. 15363416

[30] Yin C, Kiskowski M, Pouille PA, Farge E, and Solnica-Krezel L (2008). Cooperation of polarized cell intercalations drives convergence and extension of presomitic mesoderm during zebrafish gastrulation. J Cell Biol 180, 221–232. 10.1083/jcb.200704150. 18195109 PMC2213609

[31] Yen WW, Williams M, Periasamy A, Conaway M, Burdsal C, Keller R, Lu X, and Sutherland A (2009). PTK7 is essential for polarized cell motility and convergent extension during mouse gastrulation. Development 136, 2039–2048. 10.1242/dev.030601. 19439496 PMC2685725

[32] Kwon GS, Viotti M, and Hadjantonakis AK (2008). The endoderm of the mouse embryo arises by dynamic widespread intercalation of embryonic and extraembryonic lineages. Dev Cell 15, 509–520. 10.1016/j.devcel.2008.07.017. 18854136 PMC2677989

[33] Dush MK, and Nascone-Yoder NM (2019). Vangl2 coordinates cell rearrangements during gut elongation. Dev Dyn 248, 569–582. 10.1002/dvdy.61. 31081963 PMC6602813

[34] Kesavan G, Sand FW, Greiner TU, Johansson JK, Kobberup S, Wu X, Brakebusch C, and Semb H (2009). Cdc42-mediated tubulogenesis controls cell specification. Cell 139, 791–801. 10.1016/j.cell.2009.08.049. 19914171

[35] Villasenor A, Chong DC, Henkemeyer M, and Cleaver O (2010). Epithelial dynamics of pancreatic branching morphogenesis. Development 137, 4295–4305. 10.1242/dev.052993. 21098570 PMC2990215

[36] Driver EC, Northrop A, and Kelley MW (2017). Cell migration, intercalation and growth regulate mammalian cochlear extension. Development 144, 3766–3776. 10.1242/dev.151761. 28870992 PMC5675446

[37] Yang Z, Zimmerman S, Brakeman PR, Beaudoin GM 3rd, Reichardt LF, and Marciano DK (2013). De novo lumen formation and elongation in the developing nephron: a central role for afadin in apical polarity. Development 140, 1774–1784. 10.1242/dev.087957. 23487309 PMC3621492

[38] Lee JD, and Anderson KV (2008). Morphogenesis of the node and notochord: The cellular basis for the establishment and maintenance of left-right asymmetry in the mouse. Dev Dyn 237, 3464–3476. 10.1002/dvdy.21598. 18629866 PMC2593123

[39] Balmer S, Nowotschin S, and Hadjantonakis AK (2016). Notochord morphogenesis in mice: Current understanding & open questions. Dev Dyn 245, 547–557. 10.1002/dvdy.24392. 26845388 PMC4844759

[40] Sutherland A, Keller R, and Lesko A (2020). Convergent extension in mammalian morphogenesis. Semin Cell Dev Biol 100, 199–211. 10.1016/j.semcdb.2019.11.002. 31734039 PMC7071967

[41] Blum M, Andre P, Muders K, Schweickert A, Fischer A, Bitzer E, Bogusch S, Beyer T, van Straaten HW, and Viebahn C (2007). Ciliation and gene expression distinguish between node and posterior notochord in the mammalian embryo. Differentiation 75, 133–146. 10.1111/j.1432-0436.2006.00124.x. 17316383

[42] Shiratori H, and Hamada H (2006). The left-right axis in the mouse: From origin to morphology. Development 133, 2095–2104. 10.1242/dev.02384. 16672339

[43] Jessell TM (2000). Neuronal specification in the spinal cord: Inductive signals and transcriptional codes. Nat Rev Genet 1, 20–29. 10.1038/35049541. 11262869

[44] Williams M, Burdsal C, Periasamy A, Lewandoski M, and Sutherland A (2012). Mouse primitive streak forms in situ by initiation of epithelial to mesenchymal transition without migration of a cell population. Dev Dyn 241, 270–283. 10.1002/dvdy.23711. 22170865 PMC3266444

[45] Ramkumar N, Omelchenko T, Silva-Gagliardi NF, McGlade CJ, Wijnholds J, and Anderson KV (2016). Crumbs2 promotes cell ingression during the epithelial-to-mesenchymal transition at gastrulation. Nat Cell Biol 18, 1281–1291. 10.1038/ncb3442. 27870829 PMC5268168

[46] Francou A, Anderson KV, and Hadjantonakis A-K (2022). A ratchet-like apical constriction drives cell ingression during the mouse gastrulation EMT. bioRxiv, 2022.2004.2030.489707. 10.1101/2022.04.30.489707. PMC10171865 37162187

[47] Jurand A (1974). Some aspects of the development of the notochord in mouse embryos. J Embryol Exp Morphol 32, 1–33. 4141719

[48] Poelmann RE (1981). The head-process and the formation of the definitive endoderm in the mouse embryo. Anat Embryol (Berl) 162, 41–49. 10.1007/BF00318093. 7283172

[49] Sulik K, Dehart DB, Iangaki T, Carson JL, Vrablic T, Gesteland K, and Schoenwolf GC (1994). Morphogenesis of the murine node and notochordal plate. Dev Dyn 201, 260–278. 10.1002/aja.1002010309. 7881129

[50] Lee JD, Migeotte I, and Anderson KV (2010). Left-right patterning in the mouse requires Epb4.1l5-dependent morphogenesis of the node and midline. Dev Biol 346, 237–246. 10.1016/j.ydbio.2010.07.029. 20678497 PMC3141284

[51] Beddington RS (1994). Induction of a second neural axis by the mouse node. Development 120, 613–620. 10.1242/dev.120.3.613. 8162859

[52] Yamanaka Y, Tamplin OJ, Beckers A, Gossler A, and Rossant J (2007). Live imaging and genetic analysis of mouse notochord formation reveals regional morphogenetic mechanisms. Dev Cell 13, 884–896. 10.1016/j.devcel.2007.10.016. 18061569

[53] Imuta Y, Koyama H, Shi D, Eiraku M, Fujimori T, and Sasaki H (2014). Mechanical control of notochord morphogenesis by extra-embryonic tissues in mouse embryos. Mech Dev 132, 44–58. 10.1016/j.mod.2014.01.004. 24509350

[54] Koyama H, and Fujimori T (2020). Isotropic expansion of external environment induces tissue elongation and collective cell alignment. J Theor Biol 496, 110248. 10.1016/j.jtbi.2020.110248. 32275986

[55] Tam PP, Steiner KA, Zhou SX, and Quinlan GA (1997). Lineage and functional analyses of the mouse organizer. Cold Spring Harb Symp Quant Biol 62, 135–144. 9598345

[56] Kinder SJ, Tsang TE, Wakamiya M, Sasaki H, Behringer RR, Nagy A, and Tam PP (2001). The organizer of the mouse gastrula is composed of a dynamic population of progenitor cells for the axial mesoderm. Development 128, 3623–3634. 10.1242/dev.128.18.3623. 11566865

[57] Foote HP, Sumigray KD, and Lechler T (2013). FRAP analysis reveals stabilization of adhesion structures in the epidermis compared to cultured keratinocytes. PLoS One 8, e71491. 10.1371/journal.pone.0071491. 23977053 PMC3747223

[58] Wilkinson DG, Bhatt S, and Herrmann BG (1990). Expression pattern of the mouse T gene and its role in mesoderm formation. Nature 343, 657–659. 10.1038/343657a0. 1689462

[59] Muzumdar MD, Tasic B, Miyamichi K, Li L, and Luo L (2007). A global double-fluorescent Cre reporter mouse. Genesis 45, 593–605. 10.1002/dvg.20335. 17868096

[60] Kwon GS, and Hadjantonakis AK (2009). Transthyretin mouse transgenes direct RFP expression or Cre-mediated recombination throughout the visceral endoderm. Genesis 47, 447–455. 10.1002/dvg.20522. 19415627 PMC2878311

[61] Piliszek A, Kwon GS, and Hadjantonakis AK (2011). Ex utero culture and live imaging of mouse embryos. Methods Mol Biol 770, 243–257. 10.1007/978-1-61779-210-6_9. 21805267 PMC3298811

[62] Omelchenko T, Hall A, and Anderson KV (2020). β-Pix-dependent cellular protrusions propel collective mesoderm migration in the mouse embryo. Nat Commun 11, 6066. 10.1038/s41467-020-19889-1. 33247143 PMC7695707

[63] Aono S, Legouis R, Hoose WA, and Kemphues KJ (2004). PAR-3 is required for epithelial cell polarity in the distal spermatheca of C. elegans. Development 131, 2865–2874. 10.1242/dev.01146. 15151982

[64] Castelli M, Boca M, Chiaravalli M, Ramalingam H, Rowe I, Distefano G, Carroll T, and Boletta A (2013). Polycystin-1 binds Par3/aPKC and controls convergent extension during renal tubular morphogenesis. Nat Commun 4, 2658. 10.1038/ncomms3658. 24153433 PMC3967097

[65] McCaffrey LM, and Macara IG (2009). The Par3/aPKC interaction is essential for end bud remodeling and progenitor differentiation during mammary gland morphogenesis. Genes Dev 23, 1450–1460. 10.1101/gad.1795909. 19528321 PMC2701573

[66] Buckley CE, Ren X, Ward LC, Girdler GC, Araya C, Green MJ, Clark BS, Link BA, and Clarke JD (2013). Mirror-symmetric microtubule assembly and cell interactions drive lumen formation in the zebrafish neural rod. EMBO J 32, 30–44. 10.1038/emboj.2012.305. 23202854 PMC3545300

[67] Symonds AC, Buckley CE, Williams CA, and Clarke JDW (2020). Coordinated assembly and release of adhesions builds apical junctional belts during de novo polarisation of an epithelial tube. Development 147. 10.1242/dev.191494. PMC7774892 33361092

[68] Denker E, Bocina I, and Jiang D (2013). Tubulogenesis in a simple cell cord requires the formation of bi-apical cells through two discrete Par domains. Development 140, 2985–2996. 10.1242/dev.092387. 23760958

[69] Horikoshi Y, Suzuki A, Yamanaka T, Sasaki K, Mizuno K, Sawada H, Yonemura S, and Ohno S (2009). Interaction between PAR-3 and the aPKC-PAR-6 complex is indispensable for apical domain development of epithelial cells. J Cell Sci 122, 1595–1606. 10.1242/jcs.043174. 19401335

[70] Bryant DM, Datta A, Rodriguez-Fraticelli AE, Peranen J, Martin-Belmonte F, and Mostov KE (2010). A molecular network for de novo generation of the apical surface and lumen. Nat Cell Biol 12, 1035–1045. 10.1038/ncb2106. 20890297 PMC2975675

[71] Hao Y, Du Q, Chen X, Zheng Z, Balsbaugh JL, Maitra S, Shabanowitz J, Hunt DF, and Macara IG (2010). Par3 controls epithelial spindle orientation by aPKC-mediated phosphorylation of apical Pins. Curr Biol 20, 1809–1818. 10.1016/j.cub.2010.09.032. 20933426 PMC2963683

[72] Hirose T, Karasawa M, Sugitani Y, Fujisawa M, Akimoto K, Ohno S, and Noda T (2006). PAR3 is essential for cyst-mediated epicardial development by establishing apical cortical domains. Development 133, 1389–1398. 10.1242/dev.02294. 16510507

[73] Afonso C, and Henrique D (2006). PAR3 acts as a molecular organizer to define the apical domain of chick neuroepithelial cells. J Cell Sci 119, 4293–4304. 10.1242/jcs.03170. 17003110

[74] Liu WA, Chen S, Li Z, Lee CH, Mirzaa G, Dobyns WB, Ross ME, Zhang J, and Shi SH (2018). PARD3 dysfunction in conjunction with dynamic HIPPO signaling drives cortical enlargement with massive heterotopia. Genes Dev 32, 763–780. 10.1101/gad.313171.118. 29899142 PMC6049519

[75] Hayashi S, Lewis P, Pevny L, and McMahon AP (2002). Efficient gene modulation in mouse epiblast using a Sox2Cre transgenic mouse strain. Mech Dev 119 Suppl 1, S97–S101. 10.1016/s0925-4773(03)00099-6. 14516668

[76] Schluter MA, and Margolis B (2009). Apical lumen formation in renal epithelia. J Am Soc Nephrol 20, 1444–1452. 10.1681/ASN.2008090949. 19497970

[77] Datta A, Bryant DM, and Mostov KE (2011). Molecular regulation of lumen morphogenesis. Curr Biol 21, R126–136. 10.1016/j.cub.2010.12.003. 21300279 PMC3771703

[78] Blasky AJ, Mangan A, and Prekeris R (2015). Polarized protein transport and lumen formation during epithelial tissue morphogenesis. Annu Rev Cell Dev Biol 31, 575–591. 10.1146/annurev-cellbio-100814-125323. 26359775 PMC4927002

[79] Goldstein B, and Macara IG (2007). The PAR proteins: fundamental players in animal cell polarization. Dev Cell 13, 609–622. 10.1016/j.devcel.2007.10.007. 17981131 PMC2964935

[80] Nance J, and Zallen JA (2011). Elaborating polarity: PAR proteins and the cytoskeleton. Development 138, 799–809. 10.1242/dev.053538. 21303844 PMC3035085

[81] Lin D, Edwards AS, Fawcett JP, Mbamalu G, Scott JD, and Pawson T (2000). A mammalian PAR-3-PAR-6 complex implicated in Cdc42/Rac1 and aPKC signalling and cell polarity. Nat Cell Biol 2, 540–547. 10.1038/35019582. 10934475

[82] Wodarz A, Ramrath A, Grimm A, and Knust E (2000). Drosophila atypical protein kinase C associates with Bazooka and controls polarity of epithelia and neuroblasts. J Cell Biol 150, 1361–1374. 10.1083/jcb.150.6.1361. 10995441 PMC2150710

[83] Suzuki A, Yamanaka T, Hirose T, Manabe N, Mizuno K, Shimizu M, Akimoto K, Izumi Y, Ohnishi T, and Ohno S (2001). Atypical protein kinase C is involved in the evolutionarily conserved par protein complex and plays a critical role in establishing epithelia-specific junctional structures. J Cell Biol 152, 1183–1196. 10.1083/jcb.152.6.1183. 11257119 PMC2199212

[84] Harris TJ, and Peifer M (2005). The positioning and segregation of apical cues during epithelial polarity establishment in Drosophila. J Cell Biol 170, 813–823. 10.1083/jcb.200505127. 16129788 PMC2171335

[85] Morais-de-Sa E, Mirouse V, and St Johnston D (2010). aPKC phosphorylation of Bazooka defines the apical/lateral border in Drosophila epithelial cells. Cell 141, 509–523. 10.1016/j.cell.2010.02.040. 20434988 PMC2885938

[86] Walther RF, and Pichaud F (2010). Crumbs/DaPKC-dependent apical exclusion of Bazooka promotes photoreceptor polarity remodeling. Curr Biol 20, 1065–1074. 10.1016/j.cub.2010.04.049. 20493700

[87] Hurd TW, Gao L, Roh MH, Macara IG, and Margolis B (2003). Direct interaction of two polarity complexes implicated in epithelial tight junction assembly. Nat Cell Biol 5, 137–142. 10.1038/ncb923. 12545177

[88] Lemmers C, Michel D, Lane-Guermonprez L, Delgrossi MH, Medina E, Arsanto JP, and Le Bivic A (2004). CRB3 binds directly to Par6 and regulates the morphogenesis of the tight junctions in mammalian epithelial cells. Mol Biol Cell 15, 1324–1333. 10.1091/mbc.e03-04-0235. 14718572 PMC363137

[89] Tan B, Yatim S, Peng S, Gunaratne J, Hunziker W, and Ludwig A (2020). The mammalian Crumbs complex defines a distinct polarity domain apical of epithelial tight junctions. Curr Biol 30, 2791–2804 e2796. 10.1016/j.cub.2020.05.032. 32531288

[90] Ahmed SM, and Macara IG (2017). The Par3 polarity protein is an exocyst receptor essential for mammary cell survival. Nat Commun 8, 14867. 10.1038/ncomms14867. 28358000 PMC5379108

[91] Muller HA, and Wieschaus E (1996). armadillo, bazooka, and stardust are critical for early stages in formation of the zonula adherens and maintenance of the polarized blastoderm epithelium in Drosophila. J Cell Biol 134, 149–163. 10.1083/jcb.134.1.149. 8698811 PMC2120925

[92] Harris TJ, and Peifer M (2004). Adherens junction-dependent and -independent steps in the establishment of epithelial cell polarity in Drosophila. J Cell Biol 167, 135–147. 10.1083/jcb.200406024. 15479740 PMC2172516

[93] Achilleos A, Wehman AM, and Nance J (2010). PAR-3 mediates the initial clustering and apical localization of junction and polarity proteins during C. elegans intestinal epithelial cell polarization. Development 137, 1833–1842. 10.1242/dev.047647. 20431121 PMC2867319

[94] Simoes S, Blankenship JT, Weitz O, Farrell DL, Tamada M, Fernandez-Gonzalez R, and Zallen JA (2010). Rho-kinase directs Bazooka/Par-3 planar polarity during Drosophila axis elongation. Dev Cell 19, 377–388. 10.1016/j.devcel.2010.08.011. 20833361 PMC3131216

[95] Mizuno K, Suzuki A, Hirose T, Kitamura K, Kutsuzawa K, Futaki M, Amano Y, and Ohno S (2003). Self-association of PAR-3-mediated by the conserved N-terminal domain contributes to the development of epithelial tight junctions. J Biol Chem 278, 31240–31250. 10.1074/jbc.M303593200. 12756256

[96] Chen X, and Macara IG (2005). Par-3 controls tight junction assembly through the Rac exchange factor Tiam1. Nat Cell Biol 7, 262–269. 10.1038/ncb1226. 15723052

[97] Chen X, and Macara IG (2006). Par-3 mediates the inhibition of LIM kinase 2 to regulate cofilin phosphorylation and tight junction assembly. J Cell Biol 172, 671–678. 10.1083/jcb.200510061. 16505165 PMC2063700

[98] Ooshio T, Fujita N, Yamada A, Sato T, Kitagawa Y, Okamoto R, Nakata S, Miki A, Irie K, and Takai Y (2007). Cooperative roles of Par-3 and afadin in the formation of adherens and tight junctions. J Cell Sci 120, 2352–2365. 10.1242/jcs.03470. 17606991

[99] Labbe JC, Maddox PS, Salmon ED, and Goldstein B (2003). PAR proteins regulate microtubule dynamics at the cell cortex in C. elegans. Curr Biol 13, 707–714. 10.1016/s0960-9822(03)00251-3. 12725727

[100] Schmoranzer J, Fawcett JP, Segura M, Tan S, Vallee RB, Pawson T, and Gundersen GG (2009). Par3 and dynein associate to regulate local microtubule dynamics and centrosome orientation during migration. Curr Biol 19, 1065–1074. 10.1016/j.cub.2009.05.065. 19540120 PMC2749750

[101] Feldman JL, and Priess JR (2012). A role for the centrosome and PAR-3 in the hand-off of MTOC function during epithelial polarization. Curr Biol 22, 575–582. 10.1016/j.cub.2012.02.044. 22425160 PMC3409831

[102] Chen S, Chen J, Shi H, Wei M, Castaneda-Castellanos DR, Bultje RS, Pei X, Kriegstein AR, Zhang M, and Shi SH (2013). Regulation of microtubule stability and organization by mammalian Par3 in specifying neuronal polarity. Dev Cell 24, 26–40. 10.1016/j.devcel.2012.11.014. 23273878 PMC3549028

[103] Landin Malt A, Dailey Z, Holbrook-Rasmussen J, Zheng Y, Hogan A, Du Q, and Lu X (2019). Par3 is essential for the establishment of planar cell polarity of inner ear hair cells. Proc Natl Acad Sci U S A 116, 4999–5008. 10.1073/pnas.1816333116. 30814219 PMC6421412

[104] Blankenship JT, Backovic ST, Sanny JS, Weitz O, and Zallen JA (2006). Multicellular rosette formation links planar cell polarity to tissue morphogenesis. Dev Cell 11, 459–470. 10.1016/j.devcel.2006.09.007. 17011486

[105] Nishimura T, and Takeichi M (2008). Shroom3-mediated recruitment of Rho kinases to the apical cell junctions regulates epithelial and neuroepithelial planar remodeling. Development 135, 1493–1502. 10.1242/dev.019646. 18339671

[106] Ernst S, Liu K, Agarwala S, Moratscheck N, Avci ME, Dalle Nogare D, Chitnis AB, Ronneberger O, and Lecaudey V (2012). Shroom3 is required downstream of FGF signalling to mediate proneuromast assembly in zebrafish. Development 139, 4571–4581. 10.1242/dev.083253. 23136387

[107] Harding MJ, and Nechiporuk AV (2012). Fgfr-Ras-MAPK signaling is required for apical constriction via apical positioning of Rho-associated kinase during mechanosensory organ formation. Development 139, 3130–3135. 10.1242/dev.082271. 22833124 PMC3413159

[108] Lienkamp SS, Liu K, Karner CM, Carroll TJ, Ronneberger O, Wallingford JB, and Walz G (2012). Vertebrate kidney tubules elongate using a planar cell polarity-dependent, rosette-based mechanism of convergent extension. Nat Genet 44, 1382–1387. 10.1038/ng.2452. 23143599 PMC4167614

[109] Williams M, Yen W, Lu X, and Sutherland A (2014). Distinct apical and basolateral mechanisms drive planar cell polarity-dependent convergent extension of the mouse neural plate. Dev Cell 29, 34–46. 10.1016/j.devcel.2014.02.007. 24703875 PMC4120093

[110] Houssin NS, Martin JB, Coppola V, Yoon SO, and Plageman TF Jr. (2020). Formation and contraction of multicellular actomyosin cables facilitate lens placode invagination. Dev Biol 462, 36–49. 10.1016/j.ydbio.2020.02.014. 32113830 PMC7225080

[111] Shah PK, Tanner MR, Kovacevic I, Rankin A, Marshall TE, Noblett N, Tran NN, Roenspies T, Hung J, Chen Z, (2017). PCP and SAX-3/Robo pathways cooperate to regulate convergent extension-based nerve cord assembly in C. elegans. Dev Cell 41, 195–203 e193. 10.1016/j.devcel.2017.03.024. 28441532 PMC5469364

[112] Migeotte I, Grego-Bessa J, and Anderson KV (2011). Rac1 mediates morphogenetic responses to intercellular signals in the gastrulating mouse embryo. Development 138, 3011–3020. 10.1242/dev.059766. 21693517 PMC3119308

[113] Debnath J, and Brugge JS (2005). Modelling glandular epithelial cancers in three-dimensional cultures. Nat Rev Cancer 5, 675–688. 10.1038/nrc1695. 16148884

[114] Martin-Belmonte F, Gassama A, Datta A, Yu W, Rescher U, Gerke V, and Mostov K (2007). PTEN-mediated apical segregation of phosphoinositides controls epithelial morphogenesis through Cdc42. Cell 128, 383–397. 10.1016/j.cell.2006.11.051. 17254974 PMC1865103

[115] Jaffe AB, Kaji N, Durgan J, and Hall A (2008). Cdc42 controls spindle orientation to position the apical surface during epithelial morphogenesis. J Cell Biol 183, 625–633. 10.1083/jcb.200807121. 19001128 PMC2582895

[116] Durgan J, Kaji N, Jin D, and Hall A (2011). Par6B and atypical PKC regulate mitotic spindle orientation during epithelial morphogenesis. J Biol Chem 286, 12461–12474. 10.1074/jbc.M110.174235. 21300793 PMC3069449

[117] Buckley C, and Clarke J (2014). Establishing the plane of symmetry for lumen formation and bilateral brain formation in the zebrafish neural rod. Semin Cell Dev Biol 31, 100–105. 10.1016/j.semcdb.2014.04.008. 24721474

[118] Lowery LA, and Sive H (2005). Initial formation of zebrafish brain ventricles occurs independently of circulation and requires the nagie oko and snakehead/atp1a1a.1 gene products. Development 132, 2057–2067. 10.1242/dev.01791. 15788456

[119] Bagnat M, Cheung ID, Mostov KE, and Stainier DY (2007). Genetic control of single lumen formation in the zebrafish gut. Nat Cell Biol 9, 954–960. 10.1038/ncb1621. 17632505

[120] Dumortier JG, Le Verge-Serandour M, Tortorelli AF, Mielke A, de Plater L, Turlier H, and Maitre JL (2019). Hydraulic fracturing and active coarsening position the lumen of the mouse blastocyst. Science 365, 465–468. 10.1126/science.aaw7709. 31371608

[121] Narayanan V, Schappell LE, Mayer CR, Duke AA, Armiger TJ, Arsenovic PT, Mohan A, Dahl KN, Gleghorn JP, and Conway DE (2020). Osmotic gradients in epithelial acini increase mechanical tension across E-cadherin, drive morphogenesis, and maintain homeostasis. Curr Biol 30, 624–633 e624. 10.1016/j.cub.2019.12.025. 31983640 PMC7153951

[122] Straight SW, Shin K, Fogg VC, Fan S, Liu CJ, Roh M, and Margolis B (2004). Loss of PALS1 expression leads to tight junction and polarity defects. Mol Biol Cell 15, 1981–1990. 10.1091/mbc.e03-08-0620. 14718565 PMC379292

[123] Shin K, Straight S, and Margolis B (2005). PATJ regulates tight junction formation and polarity in mammalian epithelial cells. J Cell Biol 168, 705–711. 10.1083/jcb.200408064. 15738264 PMC2171825

[124] Torkko JM, Manninen A, Schuck S, and Simons K (2008). Depletion of apical transport proteins perturbs epithelial cyst formation and ciliogenesis. J Cell Sci 121, 1193–1203. 10.1242/jcs.015495. 18349078

[125] Schluter MA, Pfarr CS, Pieczynski J, Whiteman EL, Hurd TW, Fan S, Liu CJ, and Margolis B (2009). Trafficking of Crumbs3 during cytokinesis is crucial for lumen formation. Mol Biol Cell 20, 4652–4663. 10.1091/mbc.E09-02-0137. 19776356 PMC2777096

[126] Ferrari A, Veligodskiy A, Berge U, Lucas MS, and Kroschewski R (2008). ROCK-mediated contractility, tight junctions and channels contribute to the conversion of a preapical patch into apical surface during isochoric lumen initiation. J Cell Sci 121, 3649–3663. 10.1242/jcs.018648. 18946028

[127] Vega-Salas DE, Salas PJ, and Rodriguez-Boulan E (1988). Exocytosis of vacuolar apical compartment (VAC): A cell-cell contact controlled mechanism for the establishment of the apical plasma membrane domain in epithelial cells. J Cell Biol 107, 1717–1728. 10.1083/jcb.107.5.1717. 3053735 PMC2115332

[128] Bedzhov I, and Zernicka-Goetz M (2014). Self-organizing properties of mouse pluripotent cells initiate morphogenesis upon implantation. Cell 156, 1032–1044. 10.1016/j.cell.2014.01.023. 24529478 PMC3991392

[129] Amack JD, Wang X, and Yost HJ (2007). Two T-box genes play independent and cooperative roles to regulate morphogenesis of ciliated Kupffer’s vesicle in zebrafish. Dev Biol 310, 196–210. 10.1016/j.ydbio.2007.05.039. 17765888

[130] Oteiza P, Koppen M, Concha ML, and Heisenberg CP (2008). Origin and shaping of the laterality organ in zebrafish. Development 135, 2807–2813. 10.1242/dev.022228. 18635607

[131] Navis A, Marjoram L, and Bagnat M (2013). Cftr controls lumen expansion and function of Kupffer’s vesicle in zebrafish. Development 140, 1703–1712. 10.1242/dev.091819. 23487313 PMC3621488

[132] Cote LE, and Feldman JL (2022). Won’t you be my neighbor: How epithelial cells connect together to build global tissue polarity. Front Cell Dev Biol 10, 887107. 10.3389/fcell.2022.887107. 35800889 PMC9253303

[133] Horne-Badovinac S, Lin D, Waldron S, Schwarz M, Mbamalu G, Pawson T, Jan Y, Stainier DY, and Abdelilah-Seyfried S (2001). Positional cloning of heart and soul reveals multiple roles for PKC lambda in zebrafish organogenesis. Curr Biol 11, 1492–1502. 10.1016/s0960-9822(01)00458-4. 11591316

[134] Alvers AL, Ryan S, Scherz PJ, Huisken J, and Bagnat M (2014). Single continuous lumen formation in the zebrafish gut is mediated by smoothened-dependent tissue remodeling. Development 141, 1110–1119. 10.1242/dev.100313. 24504339 PMC3929411

[135] Guo C, Zou J, Wen Y, Fang W, Stolz DB, Sun M, and Wei X (2018). Apical cell-cell adhesions reconcile symmetry and asymmetry in zebrafish neurulation. iScience 3, 63–85. 10.1016/j.isci.2018.04.007. 29901027 PMC5994761

[136] Denker E, Sehring IM, Dong B, Audisso J, Mathiesen B, and Jiang D (2015). Regulation by a TGFβ-ROCK-actomyosin axis secures a non-linear lumen expansion that is essential for tubulogenesis. Development 142, 1639–1650. 10.1242/dev.117150. 25834020

[137] Strilíc B, Kucera T, Eglinger J, Hughes MR, McNagny KM, Tsukita S, Dejana E, Ferrara N, and Lammert E (2009). The molecular basis of vascular lumen formation in the developing mouse aorta. Dev Cell 17, 505–515. 10.1016/j.devcel.2009.08.011. 19853564

[138] Harding MJ, McGraw HF, and Nechiporuk A (2014). The roles and regulation of multicellular rosette structures during morphogenesis. Development 141, 2549–2558. 10.1242/dev.101444. 24961796 PMC4067956

[139] Fan L, Kovacevic I, Heiman MG, and Bao Z (2019). A multicellular rosette-mediated collective dendrite extension. Elife 8. 10.7554/eLife.38065. PMC6400498 30767892

[140] Saykali B, Mathiah N, Nahaboo W, Racu ML, Hammou L, Defrance M, and Migeotte I (2019). Distinct mesoderm migration phenotypes in extra-embryonic and embryonic regions of the early mouse embryo. Elife 8. 10.7554/eLife.42434. PMC6450669 30950395

[141] Abboud Asleh M, Zaher M, Asleh J, Jadon J, Shaulov L, Yelin R, and Schultheiss TM (2023). A morphogenetic wave in the chick embryo lateral plate mesoderm generates mesenchymal-epithelial transition through a 3D-rosette intermediate. Dev Cell 58. 10.1016/j.devcell.2023.03.017. 37080204

[142] Wells CD, Fawcett JP, Traweger A, Yamanaka Y, Goudreault M, Elder K, Kulkarni S, Gish G, Virag C, Lim C, (2006). A Rich1/Amot complex regulates the Cdc42 GTPase and apical-polarity proteins in epithelial cells. Cell 125, 535–548. 10.1016/j.cell.2006.02.045. 16678097

[143] Stevenson BR, Siliciano JD, Mooseker MS, and Goodenough DA (1986). Identification of ZO-1: a high molecular weight polypeptide associated with the tight junction (zonula occludens) in a variety of epithelia. J Cell Biol 103, 755–766. 10.1083/jcb.103.3.755. 3528172 PMC2114282

[144] Schindelin J, Arganda-Carreras I, Frise E, Kaynig V, Longair M, Pietzsch T, Preibisch S, Rueden C, Saalfeld S, Schmid B, (2012). Fiji: an open-source platform for biological-image analysis. Nature Methods 9, 676–682. 10.1038/nmeth.2019. 22743772 PMC3855844

[145] Preibisch S, Saalfeld S, and Tomancak P (2009). Globally optimal stitching of tiled 3D microscopic image acquisitions. Bioinformatics 25, 1463–1465. 10.1093/bioinformatics/btp184. 19346324 PMC2682522

[146] Cardona A, Saalfeld S, Schindelin J, Arganda-Carreras I, Preibisch S, Longair M, Tomancak P, Hartenstein V, and Douglas RJ (2012). TrakEM2 software for neural circuit reconstruction. PLoS One 7, e38011. 10.1371/journal.pone.0038011. 22723842 PMC3378562

[147] Lawson KA, and Wilson V (2016). A Revised Staging of Mouse Development Before Organogenesis. In Kaufman’s Atlas of Mouse Development Supplement, Baldock R, Bard J, Davidson DR, and Morriss-Kay G, eds. (Academic Press), pp. 51–64. 10.1016/B978-0-12-800043-4.00003-8.

[148] Downs KM, and Davies T (1993). Staging of gastrulating mouse embryos by morphological landmarks in the dissecting microscope. Development 118, 1255–1266. 10.1242/dev.118.4.1255. 8269852

[149] Nieto MA, Patel K, and Wilkinson DG (1996). In situ hybridization analysis of chick embryos in whole mount and tissue sections. In Methods in Cell Biology, Bronner-Fraser M, ed. (Academic Press), pp. 219–235. 10.1016/S0091-679X(08)60630-5. 8722478

