# Supplementary material for: Multicellular rosettes link mesenchymal-epithelial transition to radial intercalation in the mouse axial mesoderm
Source: Dev Cell. Author manuscript; Available in PMC 2023 Jun 8. (PMC10247533; doi:10.1016/j.devcel.2023.03.018)
Supplement: Supplement — Table S1. Summary of data and statistical analyses. Related to Figures 1–7 and S1–S7. [file NIHMS1892904-supplement-Supplement.pdf]

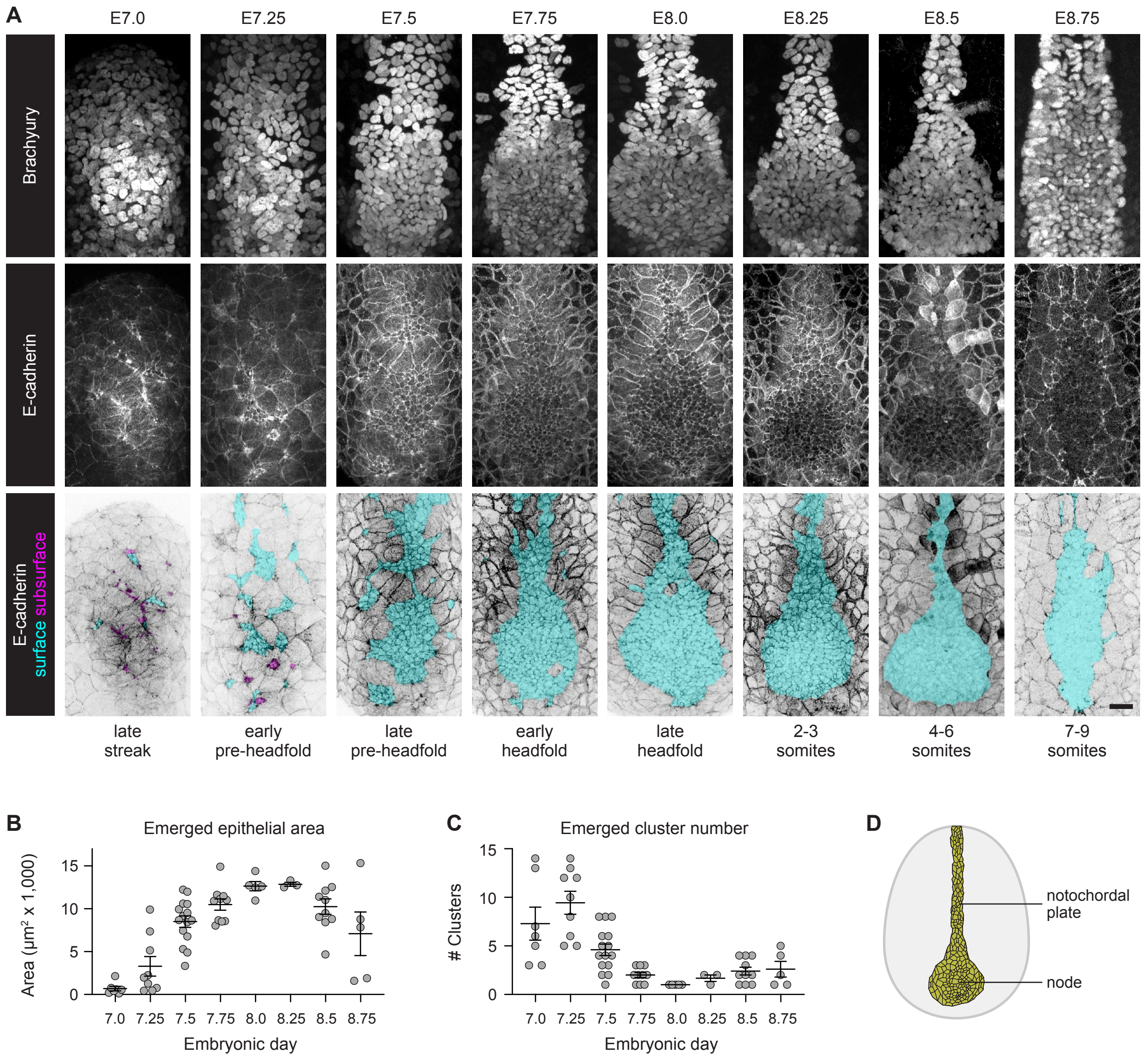

Figure S1. Emergence and internalization of the axial mesoderm epithelium. Related to Figure 1.

(A) Axial mesoderm cells in wild-type mouse embryos visualized with Brachyury (white, top row) and E-cadherin (white, middle row; black, bottom row). Emerged cells (cyan), unemerged cells (magenta). Ventral views, anterior up (maximum-intensity projections). Bar, 25 μm.

(B and C) Emerged apical area (B) and number of emerged clusters (C) in the axial mesoderm epithelium. Data for E7.0-E8.0 are reproduced from Figure 1. The axial mesoderm begins to submerge at E8.5 to form the notochord. Each dot indicates one embryo (3-15 embryos/stage). Mean±SEM between embryos is shown.

(D) Schematic of the E8.0 node (also known as the ventral node or posterior notochord) and notochordal plate.

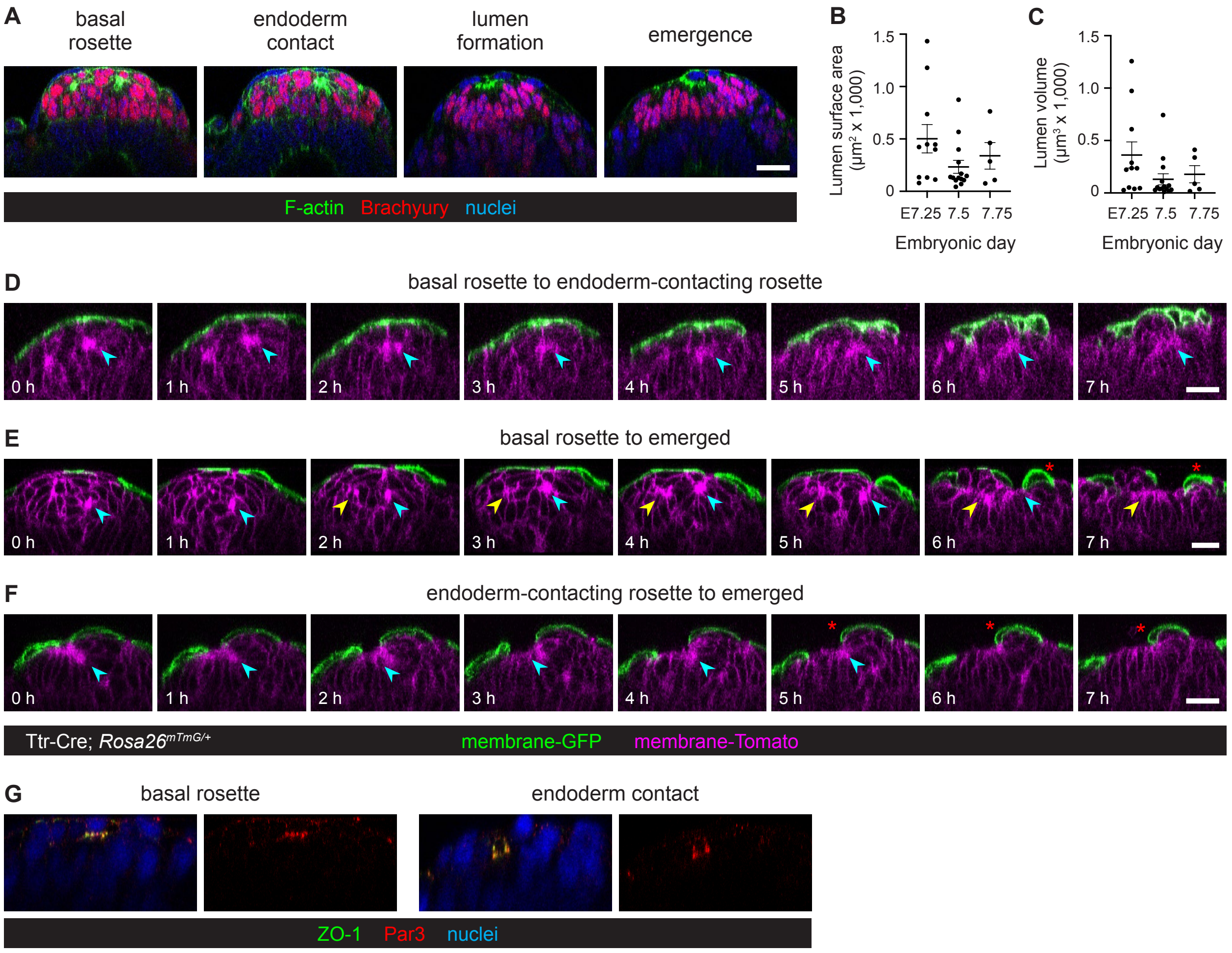

Figure S2. Protein localization, lumen formation, and dynamic behaviors in axial mesoderm rosettes. Related to Figure 3.

(A) Localization of F-actin (phalloidin, green), Brachyury (red), and nuclei (Hoechst, blue) in axial mesoderm rosettes. Two basal rosettes are shown in the left panel.

(B and C) Lumen surface area (B) and volume (C) in control embryos. No significant differences between stages were observed (unpaired t-test). Each dot indicates one lumen. Mean±SEM between lumens is shown.

(D-F) Stills from time-lapse imaging of Ttr-Cre; *Rosa26*$^{mTmG/+}$ embryos. Visceral endoderm cells (green), axial mesoderm and non-visceral endoderm cells (magenta). Rosettes (noted with cyan and yellow arrowheads) translocate toward the embryo surface, shown progressing from basal to endoderm-contacting (D), basal to emerged (E), and endoderm-contacting to emerged (F). Two rosettes fuse together before emerging in E. Endoderm cells adopt curved morphologies (red asterisks) as they interact with rosettes during emergence. n=17 rosettes in 7 embryos.

(G) Localization of ZO-1 (green), Par3 (red), and nuclei (Hoechst, blue) in axial mesoderm rosettes. Left panels show ZO-1, Par3, and nuclei. Right panels show Par3 alone.

Optically reconstructed transverse views, apical up. Bars, 25 µm.

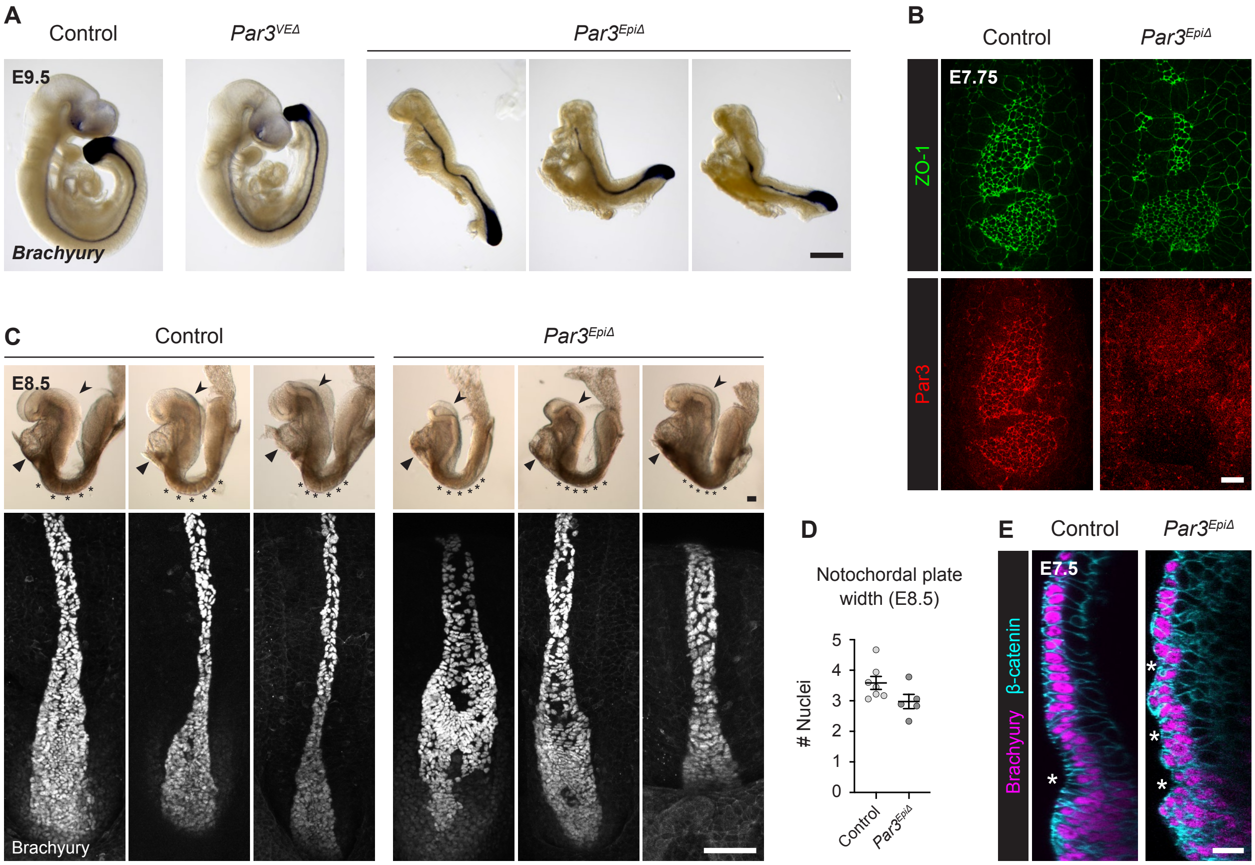

Figure S3. Notochord differentiation, convergence, and organization in *Par3* mutant embryos. Related to Figure 4.

(A) Whole mount *in situ* hybridization with a *Brachyury* riboprobe in E9.5 control, *Par3*$^{VE\Delta}$, and *Par3*$^{Epi\Delta}$ embryos. Brachyury is expressed in the notochord, which extends along the anterior-posterior axis, and terminates in a broad, diffuse posterior domain in the tailbud. Lateral views, anterior up. Bar, 500 µm. n = 13 control, 4 *Par3*$^{VE\Delta}$ mutant, and 7 *Par3*$^{Epi\Delta}$ mutant embryos.

(B) Maximum-intensity projections of confocal z-stacks of E7.75 control and *Par3*$^{Epi\Delta}$ embryos. ZO-1 (green), Par3 (red). Ventral views, anterior up. Bar, 25 µm.

(C) Light micrographs (top) and maximum-intensity projections of confocal z-stacks (bottom) of E8.5 control and *Par3*$^{Epi\Delta}$ mutant embryos. Brachyury (white). The somites (asterisks), headfolds (arrowheads), and heart (triangles) are indicated. Top, lateral views, anterior left. Bottom, ventral views, anterior up. Bars, 100 µm.

(D) The number of Brachyury-positive nuclei across the mediolateral width of the notochordal plate is not significantly different between control and *Par3*$^{Epi\Delta}$ mutant embryos at E8.5 (p=0.09, unpaired t-test). Each dot shows the average of 7 measurements/embryo. Mean±SEM between embryos is shown.

(E) Optically reconstructed sagittal views of views (anterior up) in E7.5 control and *Par3*$^{Epi\Delta}$ mutant embryos. Brachyury (magenta), β-catenin (cyan). Asterisks, surface depressions in the axial mesoderm. Bar, 25 µm.

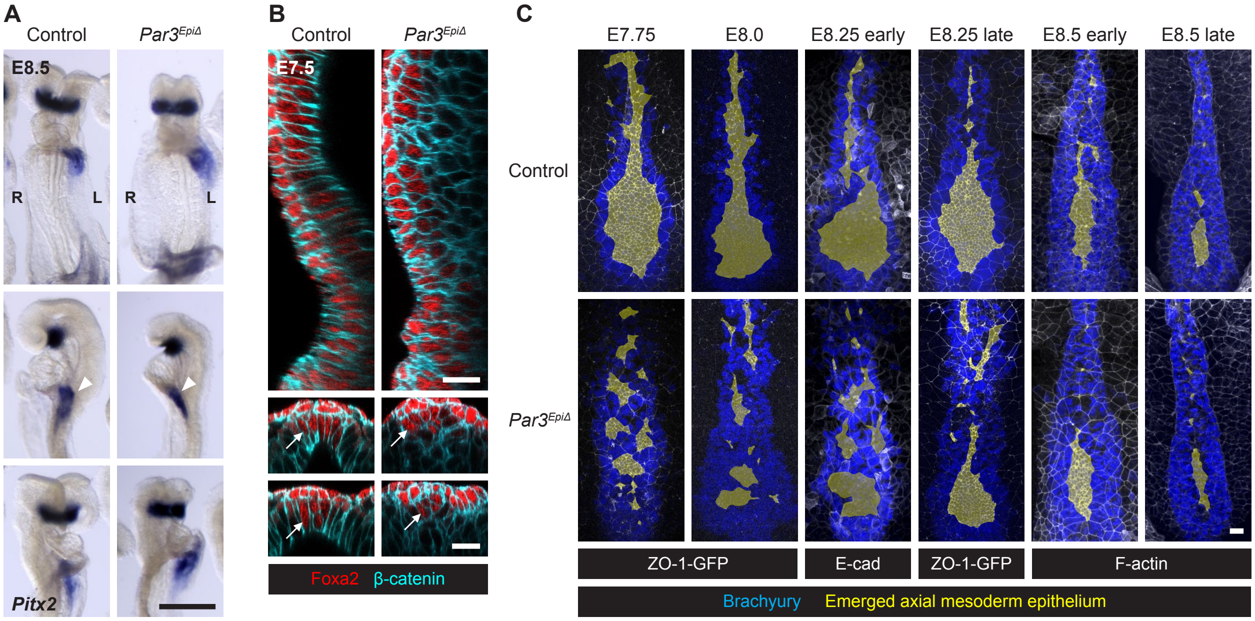

Figure S4. Axial mesoderm signaling and epithelial sheet formation in *Par3* mutant embryos. Related to Figure 4.

(A) Whole mount *in situ* hybridization with a *Pitx2* riboprobe in E8.5 control and *Par3*^{EpiΔ} mutant embryos. *Pitx2* transcripts are evident in the left lateral plate mesoderm (white triangles) and no expression is visible in the right lateral plate mesoderm in both control and *Par3*^{EpiΔ} mutant embryos, indicating that left/right asymmetry is established in *Par3*^{EpiΔ} mutant embryos. Top, ventral views. Middle, left views. Bottom, oblique right views. Anterior up in all panels. Bar, 500 µm. n = 10 control and 6 *Par3*^{EpiΔ} embryos.

(B) Optically reconstructed sagittal (top) and transverse (middle and bottom) views of E7.5 control and *Par3*^{EpiΔ} mutant embryos. Foxa2 (red), β-catenin (cyan). Foxa2-positive nuclei are present in the neural plate (white arrows), adjacent to the axial mesoderm, in control and *Par3*^{EpiΔ} mutant embryos, indicating floor plate specification. Note the aberrant multilayered morphology of the neural plate in *Par3*^{EpiΔ} mutants. Bars, 25 µm.

(C) Maximum-intensity projections of Brachyury (blue) and ZO-1-GFP, E-cadherin, or F-actin (white) in control and *Par3*^{EpiΔ} mutant embryos through a stage series spanning the conclusion of emergence (E7.75 and 8.0) and partially through submergence (E8.25 - 8.5). Outlines of the emerged axial mesoderm (yellow) highlight the morphology of the epithelial sheet. In control embryos at stages after emergence, the axial mesoderm shifts from a continuous to discontinuous sheet, whereas the axial mesoderm of *Par3*^{EpiΔ} mutant embryos is fragmented throughout. Ventral views, anterior up. Bar, 25 µm.

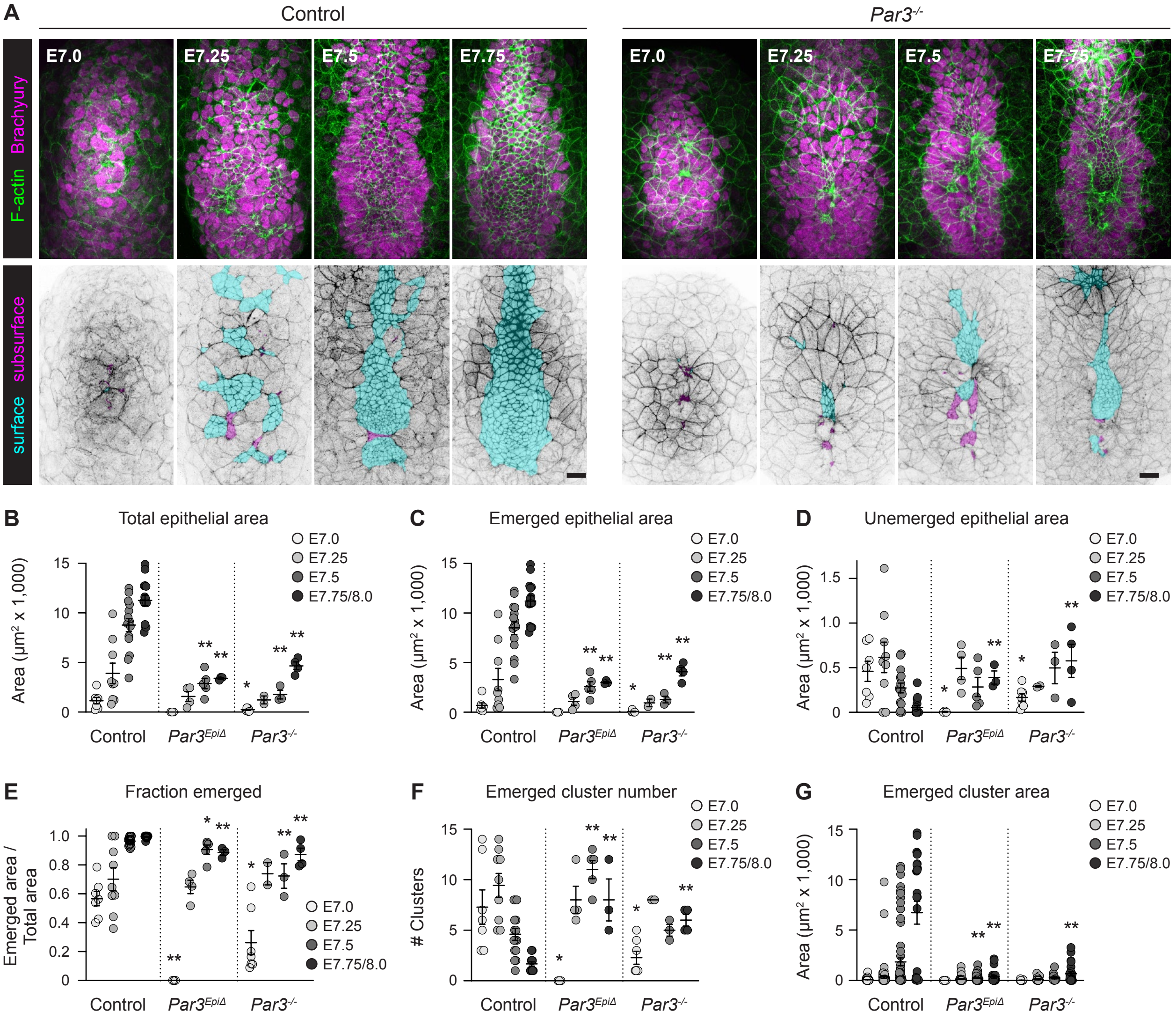

Figure S5. Axial mesoderm organization in *Par3* null mutants. Related to Figure 4.

(A) Localization of Brachyury (magenta, top panels) and F-actin (green, top panels; black, bottom panels) in control and *Par3*–/– null mutant embryos. Emerged cells (cyan), unemerged cells (magenta) (maximum-intensity projections).

(B) Total apical area of the axial mesoderm epithelium (emerged and unemerged regions) at the indicated stages in control, $Par3^{Epi\Delta}$, and *Par3*–/– null mutant embryos.

(C and D) Emerged apical area (C) and unemerged apical area (D) in control, $Par3^{Epi\Delta}$, and *Par3*–/– null mutant embryos.

(E) Fraction of the total apical area of the axial mesoderm epithelium that is located on the surface at the indicated stages in control, $Par3^{Epi\Delta}$, and *Par3*–/– null mutant embryos.

(F and G) Emerged cluster area (F) and number of emerged clusters (G) in control, $Par3^{Epi\Delta}$, and *Par3*–/– null mutant embryos.

Ventral views, anterior up. Each dot indicates one embryo (B-F) or cluster (G), 2-15 embryos per stage for each genotype. Mean±SEM between embryos (B-F) or clusters (G) is shown. $*p<0.04$, $**p\leq0.0003$ compared to controls (unpaired t-test). Controls in B-G show combined data for $Par3^{Epi\Delta}$ and *Par3*–/– littermate controls. Some data are reproduced from Figure 4. Bars, 25 µm.

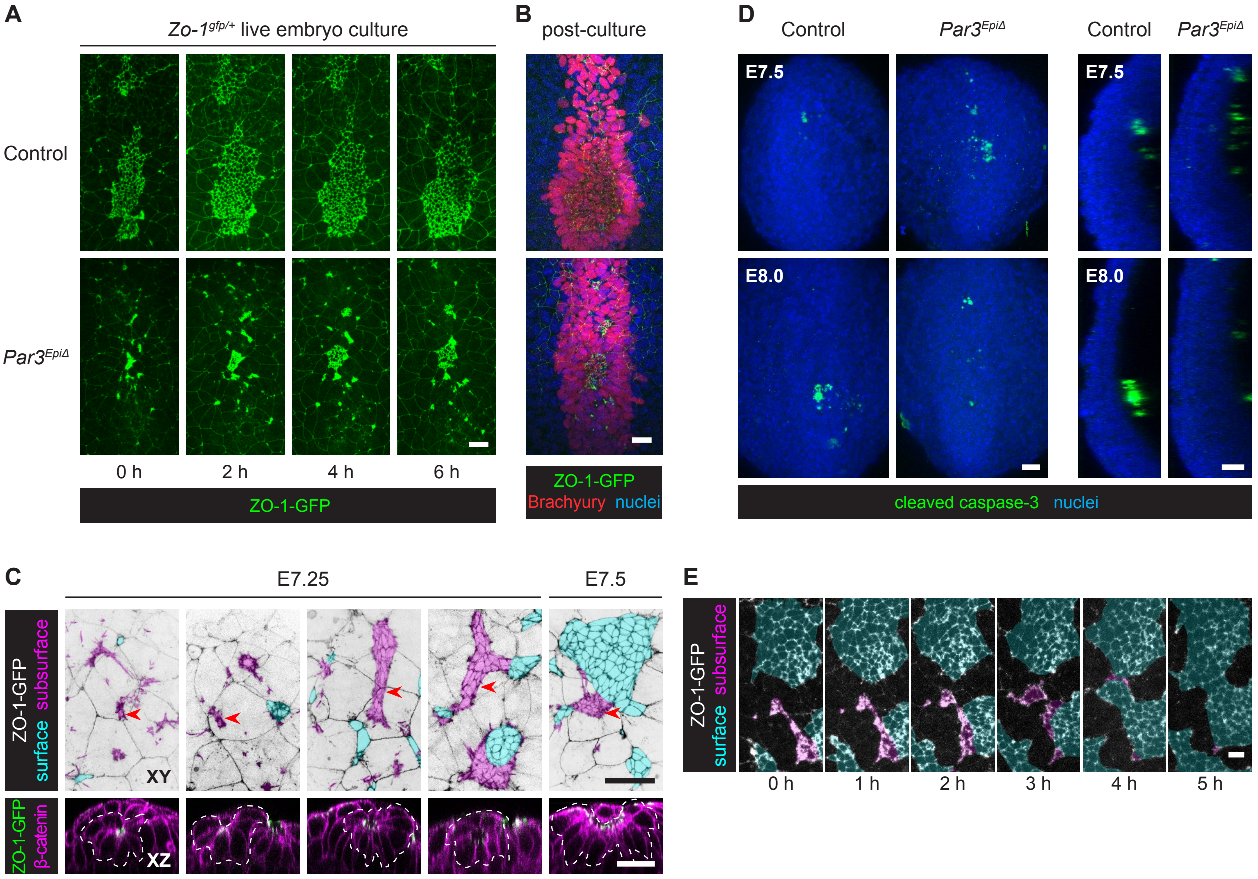

Figure S6. ZO-1-GFP localization, cell death, and rosette dynamics in fixed and live embryos. Related to Figure 5.

(A) Stills from a time-lapse movie of control and *Par3^{EpiΔ}* mutant embryos expressing ZO-1-GFP (green).

(B) Embryos fixed immediately after imaging showing ZO-1-GFP (green), Brachyury (red), and nuclei (Hoechst, blue).

(C) Localization of ZO-1-GFP (black) in fixed embryos. Emerged cells (cyan), unemerged cells (magenta). Red arrowheads indicate the planes shown in transverse views. Bottom panels, optically reconstructed transverse views. ZO-1-GFP (green) and β-catenin (magenta). Top panels are also shown in Figure 2D.

(D) Localization of cleaved caspase-3 (green) in control and *Par3^{EpiΔ}* mutant embryos at E7.5 and 8.0. Enrichment of cleaved caspase-3 is most abundant in apoptotic cells housed within the amniotic cavity and distinct from the embryo proper, visible in 25 μm optically reconstructed sagittal views (right panels). Left panels, maximum-intensity projections.

(E) Stills from a time-lapse movie of a control embryo expressing ZO-1-GFP (white). Two axial mesoderm clusters coalesce through a subsurface tunnel.

Ventral views, anterior up (maximum-intensity projections) in A, B, C (top panels), D (left panels), and E. Transverse views, apical up in C (bottom panels). Sagittal views, anterior up in D (right panels). Bars, 25 μm in A-D, 10 μm in E.

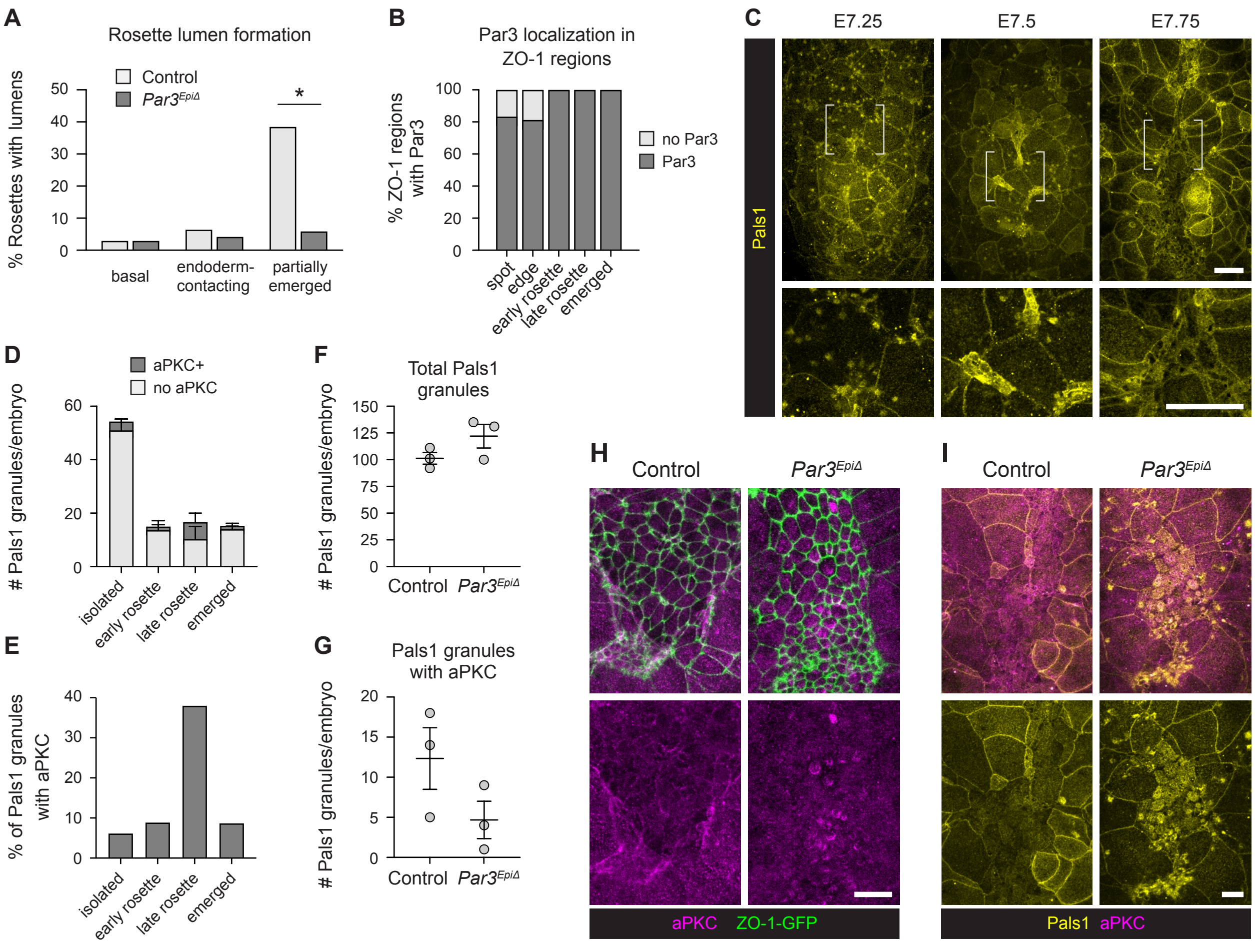

Figure S7. Pals1 localization in control and *Par3* mutant embryos. Related to Figure 7.

(A) Percentage of basal, endoderm-contacting, and partially emerged rosettes with and without lumens in control and *Par3*$^{Epi\Delta}$ mutant embryos.

(B) Par3 localization in the indicated ZO-1-GFP regions in control embryos.

(C) Localization of Pals1 (yellow) during axial mesoderm emergence. Brackets in top panels denote image regions shown in bottom panels.

(D and E) Number of Pals1 granules with and without aPKC (D) and percentage of Pals1 granules with aPKC (E) in the indicated ZO-1-GFP regions in control embryos.

(F and G) Number of Pals1 granules (F) and aPKC-positive Pals1 granules (G) in control and *Par3*$^{Epi\Delta}$ mutant embryos. Each dot indicates one embryo.

(H) Maximum-intensity projections showing aPKC (magenta, top and bottom panels) and ZO-1-GFP (green, top panels) in emerged axial mesoderm cells in control and *Par3*$^{Epi\Delta}$ mutant embryos.

(I) Maximum-intensity projections showing Pals1 (yellow, top and bottom panels) and aPKC (magenta, top panels) in emerged axial mesoderm cells in control and *Par3*$^{Epi\Delta}$ mutant embryos.

Mean±SEM between structures (D) or embryos (F and G) is shown, * p=0.0008 compared to controls (Fisher's exact test in A, unpaired t-test in F and G). 117 rosettes in 23 control embryos and 92 rosettes in 14 *Par3*$^{Epi\Delta}$ mutant embryos at E7.25-E7.75 in A, 89 ZO-1-GFP regions in 7 control embryos in B, and 304 granules in 3 control embryos and 366 granules in 3 *Par3*$^{Epi\Delta}$ mutants in D-G. Bars, 25 µm.

Table S1. Summary of data and statistical analyses. Related to Figures 1-7 and S1-S7.

EPITHELIAL ASSEMBLY AND EMERGENCE IN FIXED EMBRYOS

Epithelial area in fixed embryos

Related to Figures 1C, 2C, 4C, S1B, and S5B-S5E. Mean ± SEM per embryo, unpaired t-tests vs. Control.

Stage	Genotype (# embryos)	Epithelial area (µm^2) Total area per embryo	Total area p-value	Emerged area per embryo	Emerged area p-value	Unemerged area per embryo	Unemerged area p-value	Fraction emerged per embryo	Fraction emerged p-value
E7.0	Control (7)	1,148 ± 321	--	688 ± 257	--	460 ± 113	--	0.57 ± 0.050	--
	Par3$^{Epi\Delta}$ (3)	5 ± 5	0.0543	0 ± 0	0.1293	5 ± 5	0.0341	0.0 ± 0.0	<0.0001
	Par3$^{-/-}$ (7)	238 ± 60	0.0164	71 ± 37	0.0351	167 ± 42	0.0316	0.26 ± 0.084	0.0088
E7.25	Control (9)	3,905 ± 1,041	--	3,289 ± 1,137	--	616 ± 171	--	0.70 ± 0.078	--
	Par3$^{Epi\Delta}$ (4)	1,580 ± 500	0.1816	1,087 ± 379	0.2378	493 ± 125	0.6624	0.65 ± 0.047	0.6695
	Par3$^{-/-}$ (2)	1,228 ± 396	0.2755	938 ± 388	0.3749	290 ± 8.2	0.4105	0.74 ± 0.077	0.8367
E7.5	Control (15)	8,774 ± 658	--	8,502 ± 663	--	273 ± 55	--	0.97 ± 0.0073	--
	Par3$^{Epi\Delta}$ (5)	2,883 ± 531	0.0001	2,599 ± 477	0.0001	284 ± 107	0.9200	0.91 ± 0.032	0.0143
	Par3$^{-/-}$ (3)	1,770 ± 446	0.0003	1,273 ± 328	0.0002	497 ± 176	0.1375	0.72 ± 0.084	<0.0001
E7.75 – 8.0	Control (15)	11,258 ± 535	--	11,205 ± 539	--	53 ± 25	--	0.99 ± 0.0026	--
	Par3$^{Epi\Delta}$ (3)	3,402 ± 97	<0.0001	3,012 ± 102	<0.0001	390 ± 73	<0.0001	0.89 ± 0.020	<0.0001
	Par3$^{-/-}$ (4)	4,664 ± 386	<0.0001	4,087 ± 434	<0.0001	577 ± 184	<0.0001	0.87 ± 0.043	<0.0001
E7.75	Control (10)			10,488 ± 664				1.00 ± 0.0037	
E8.0	Control (5)			12,639 ± 545				0.99 ± 0.0028	
E8.25	Control (3)			12,855 ± 234					
E8.5	Control (10)			10,256 ± 894					
E8.75	Control (5)			7,086 ± 2,534					

Cluster number and area in fixed embryos

Related to Figures 1D, 4D, 4E, S1C, S5F, and S5G. Mean ± SEM per embryo or per cluster, unpaired t-tests vs. Control.

Stage	Genotype (# embryos)	# Emerged clusters n	# Emerged clusters per embryo	# Emerged clusters p-value	Emerged cluster area (µm^2) per cluster	Emerged cluster area p-value
E7.0	Control (7)	51	7.3 ± 1.7	--	94 ± 17.7	--
	Par3$^{Epi\Delta}$ (3)	0	0.0 ± 0.0	0.0267	0 ± 0*	0.2066
	Par3$^{-/-}$ (7)	16	2.3 ± 0.6	0.0176	31 ± 11.3	0.0545
E7.25	Control (9)	85	9.4 ± 1.2	--	348 ± 138	--
	Par3$^{Epi\Delta}$ (4)	32	8.0 ± 1.4	0.4859	136 ± 51.5	0.3536
	Par3$^{-/-}$ (2)	16	8.0 ± 0.0	0.5933	117 ± 50.8	0.4730
E7.5	Control (15)	69	4.6 ± 0.6	--	1,848 ± 388	--
	Par3$^{Epi\Delta}$ (5)	55	11.0 ± 0.9	<0.0001	236 ± 44.1	0.0003
	Par3$^{-/-}$ (3)	15	5.0 ± 0.6	0.7719	255 ± 93.0	0.0604
E7.75 – 8.0	Control (15)	25	1.7 ± 0.2	--	6,723 ± 1,139	--
	Par3$^{Epi\Delta}$ (3)	24	8.0 ± 2.1	<0.0001	376 ± 129	<0.0001
	Par3$^{-/-}$ (4)	24	6.0 ± 0.6	<0.0001	681 ± 189	<0.0001
E7.75	Control (10)		2.0 ± 0.3			
E8.0	Control (5)		1.0 ± 0.0			
E8.25	Control (3)		1.7 ± 0.3			
E8.5	Control (10)		2.4 ± 0.4			
E8.75	Control (5)		2.6 ± 0.8			

*Embryos with no emerged clusters were scored as having a single cluster with an area of 0 µm^2.

Emerged cell number and area in Control and *Par3*$^{Epi\Delta}$ embryos

Related to Figures 4F and 4G. Mean ± SEM per embryo or per cluster, unpaired t-tests vs. Control.

Stage	Genotype	# Emerged cells: # Embryos	Cell number range	Emerged cells per embryo	p-value	Emerged cell area: # Embryos	# Clusters	Average cell area per cluster (µm^2)	p-value
E7.5	Control	10	246 – 564	346 ± 35	0.0242	10	17	21.1 ± 1.7	< 0.0001
	Par3$^{Epi\Delta}$	7	117 – 294	231 ± 23		7	13	9.9 ± 1.0	

Width of notochordal plate in Control and *Par3*$^{Epi\Delta}$ embryos

Related to Figure S3D. Mean ± SEM per embryo, unpaired t-tests vs. Control.

Stage	Genotype (# embryos)	Width of notochordal plate in # of nuclei per embryo	p-value
E8.5	Control (7)	3.6 ± 0.2	0.0868
	Par3$^{Epi\Delta}$ (5)	3.0 ± 0.2	

ROSETTES IN FIXED AND LIVE EMBRYOS

Rosettes in Control and *Par3^EpiΔ* embryos

Related to Figures 3J, 6B, and 6C. Mean ± SEM per embryo, unpaired t-tests vs. Control.

Stage	Genotype (# embryos)	# Rosettes			Basal rosettes		Endoderm-contacting rosettes		Partially emerged rosettes	
		n	per embryo	p-value	n	per embryo	n	per embryo	n	per embryo
E7.0	Control (6)	48	8.0 ± 1.2	--	22	3.7 ± 0.4	14	2.3 ± 0.8	12	2.0 ± 0.5
E7.25	Control (8)	55	6.9 ± 1.2	0.9544	23	2.9 ± 0.9	13	1.6 ± 0.6	19	2.4 ± 0.4
	Par3^EpiΔ (3)	21	7.0 ± 0.6		12	4.0 ± 0.6	7	2.3 ± 0.3	2	0.7 ± 0.7
E7.5	Control (11)	51	4.6 ± 0.8	0.0321	9	0.8 ± 0.3	16	1.5 ± 0.3	26	2.4 ± 0.5
	Par3^EpiΔ (8)	59	7.4 ± 0.8		20	2.5 ± 0.3	13	1.6 ± 0.5	26	3.3 ± 0.7
E7.75	Control (4)	11	2.8 ± 0.5	0.3151	2	0.5 ± 0.3	2	0.5 ± 0.3	7	1.8 ± 0.5
	Par3^EpiΔ (3)	12	4.0 ± 1.2		2	0.7 ± 0.3	4	1.3 ± 0.3	6	2.0 ± 1.0

Rosette lumens in Control embryos

Related to Figure 3K.

Stage (# embryos)	Rosettes with lumens
E7.0 (6)	1/48 (2%)
E7.25 (8)	7/55 (13%)
E7.5 (11)	11/51 (22%)
E7.75 (4)	5/11 (45%)

Rosette behaviors in live-imaged Ttr-Cre; *Rosa26^mTmG/+* embryos

Related to Figures 3L and 3M, n = 17 rosettes in 7 embryos.

Initial position (# rosettes)	Ends endoderm-contacting	Ends partially emerged	Ends fully emerged
Basal (7)	3	0	4
Endoderm-contacting (5)	0	2	3
Partially emerged (5)	0	0	5

Rosette lumens in Control and *Par3^EpiΔ* embryos

Related to Figures 6C and S7A. Fisher's exact tests vs. Control.

Stage	Genotype (# embryos)	Basal rosettes		Endoderm-contacting rosettes		Partially emerged rosettes	
		with lumen	p-value	with lumen	p-value	with lumen	p-value
E7.25 – 7.75	Control (23)	1/34 (3%)	>0.9999	2/31 (6%)	>0.9999	20/52 (38%)	0.0008
	Par3^EpiΔ (14)	1/34 (3%)		1/24 (4%)		2/34 (6%)	

Rosette lumen volume and surface area in Control embryos

Related to Figures S2B and S2C. Mean ± SEM per lumen, unpaired t-tests.

Stage (# embryos)	# Lumen-containing rosettes	Surface area per lumen (μm²)	Volume per lumen (μm³)	comparison	p-value	
					surface area	volume
E7.25 (6)	11	502 ± 135	362 ± 124	E7.25 vs E7.5	0.0646	0.0762
E7.5 (7)	14	234 ± 62.1	130 ± 53.2	E7.25 vs E7.75	0.4707	0.3627
E7.75 (2)	5	339 ± 127	179 ± 81.9	E7.5 vs E7.75	0.4212	0.6404

EPITHELIAL DYNAMICS IN LIVE EMBRYOS

Pre-cluster behaviors in live-imaged Control and *Par3^EpiΔ* embryos

Related to Figure 5C. Mean ± SEM per embryo, unpaired t-tests vs. Control.

Genotype (# embryos)	# Pre-clusters			Pre-cluster behaviors: Expands			Persists			Disassembles		
	n	per embryo	p-value	n	per embryo	p-value	n	per embryo	p-value	n	per embryo	p-value
Control (5)	45	9.0 ± 2.4	0.5874	29	5.8 ± 1.9	0.0578	15	3.0 ± 0.9	0.2433	1	0.2 ± 0.2	0.1067
Par3^EpiΔ (5)	54	10.8 ± 2.1		8	1.6 ± 0.2		29	5.8 ± 2.0		17	3.4 ± 1.7	

Cluster behaviors in live-imaged Control and *Par3^EpiΔ* embryos

Related to Figure 5D. Mean ± SEM per embryo, unpaired t-tests vs. Control.

Genotype (# embryos)	# Clusters			Cluster behaviors: Emerges			Remains emerged			Does not emerge		
	n	per embryo	p-value	n	per embryo	p-value	n	per embryo	p-value	n	per embryo	p-value
Control (5)	49	9.8 ± 1.6	0.8260	13	2.6 ± 0.7	0.2725	34	6.8 ± 1.6	0.2397	2	0.4 ± 0.2	0.0140
Par3^EpiΔ (5)	47	9.4 ± 0.7		8	1.6 ± 0.5		23	4.6 ± 0.6		16	3.2 ± 0.9	

Cluster and pre-cluster coalescence events in live-imaged Control and $Par3^{Epi\Delta}$ embryos

Related to Figure 5G. Mean ± SEM per embryo, unpaired t-test vs. Control.

Genotype (# embryos)	# Coalescence events			Types of coalescence events					
				Cluster – cluster		Cluster – pre-cluster		Pre-cluster – pre-cluster	
	n	per embryo	p-value	n	per embryo	n	per embryo	n	per embryo
Control (5)	89	17.8 ± 4.6	0.0301	33	6.6 ± 1.2	39	7.8 ± 1.8	17	3.4 ± 2.7
$Par3^{Epi\Delta}$ (5)	25	5.0 ± 1.5		10	2.0 ± 0.8	10	2.0 ± 0.6	5	1.0 ± 0.5

Apical cell area after emergence in live-imaged Control and $Par3^{Epi\Delta}$ embryos

Related to Figure 5H. Mean ± SEM per cell, unpaired t-test vs. Control.

Genotype (# embryos)	# Cells	Cell area at 36 min (μm^2)		Cell area at 180 min (μm^2)	
		per cell	p-value	per cell	p-value
Control (3)	30	6.7 ± 0.6	0.0002	14.6 ± 1.2	< 0.0001
$Par3^{Epi\Delta}$ (3)	28	3.9 ± 0.3		8.1± 1.0	

PROTEIN LOCALIZATION IN FIXED EMBRYOS

aPKC localization in E7.25-E7.5 Control and $Par3^{Epi\Delta}$ embryos

Related to Figure 6E, n = 8 Control, 8 $Par3^{Epi\Delta}$ embryos. Fisher's exact tests vs. Control.

ZO-1-GFP region	Genotype	No apical aPKC	Low apical aPKC	High apical aPKC	p-value (apical vs. not apical)
Spot	Control	24/24 (100%)	0/24 (0%)	0/24 (0%)	> 0.9999
	$Par3^{Epi\Delta}$	12/12 (100%)	0/12 (0%)	0/12 (0%)	
Edge	Control	14/17 (82%)	3/17 (18%)	0/17 (0%)	0.2735
	$Par3^{Epi\Delta}$	10/10 (100%)	0/10 (0%)	0/10 (0%)	
Early rosette	Control	19/29 (66%)	8/29 (28%)	2/29 (7%)	0.0710
	$Par3^{Epi\Delta}$	13/14 (93%)	1/14 (7%)	0/14 (0%)	
Late rosette	Control	1/22 (5%)	7/22 (32%)	14/22 (64%)	< 0.0001
	$Par3^{Epi\Delta}$	15/22 (68%)	7/22 (32%)	0/22 (0%)	

Pals1 localization in E7.25-7.5 Control and $Par3^{Epi\Delta}$ embryos

Related to Figure 7E, n = 6 Control, 7 $Par3^{Epi\Delta}$ embryos. Fisher's exact tests vs. Control.

ZO-1-GFP region	Genotype	No Pals1	Granular Pals1	Transitional Pals1	Apical Pals1	p-value (apical vs. not apical)
Spot	Control	16/19 (84%)	3/19 (16%)	0/19 (0%)	0/19 (0%)	> 0.9999
	$Par3^{Epi\Delta}$	9/12 (75%)	3/12 (25%)	0/12 (0%)	0/12 (0%)	
Edge	Control	2/8 (25%)	6/8 (75%)	0/8 (0%)	0/8 (0%)	> 0.9999
	$Par3^{Epi\Delta}$	0/8 (0%)	8/8 (100%)	0/8 (0%)	0/8 (0%)	
Early rosette	Control	1/18 (6%)	14/18 (78%)	0/18 (0%)	3/18 (17%)	0.5330
	$Par3^{Epi\Delta}$	0/10 (0%)	10/10 (100%)	0/10 (0%)	0/10 (0%)	
Late rosette	Control	0/17 (0%)	5/17 (29%)	5/17 (29%)	7/17 (41%)	0.0029
	$Par3^{Epi\Delta}$	0/18 (0%)	13/18 (72%)	5/18 (28%)	0/18 (0%)	
Emerged	Control	0/22 (0%)	0/22 (0%)	8/22 (36%)	14/22 (64%)	0.0031
	$Par3^{Epi\Delta}$	0/12 (0%)	2/12 (17%)	9/12 (75%)	1/12 (8%)	

Par3 localization in Control embryos

Related to Figure S7B, n = 7 embryos.

ZO-1-GFP region	# Regions	Par3-positive regions
Spot	18	15/18 (83%)
Edge	16	13/16 (81%)
Early rosette	32	32/32 (100%)
Late rosette	14	14/14 (100%)
Emerged	9	9/9 (100%)

Pals1 granules with and without aPKC in Control embryos

Related to Figures S7D and S7E, n = 3 embryos. Mean ± SEM per embryo.

Granule type	# Pals1 granules		Pals1 granules per embryo	
	Total	aPKC-positve	aPKC-negative	aPKC-positive
Isolated	163	10 (6%)	51.0 ± 4.2	3.3 ± 0.9
Early rosette	45	4 (9%)	13.7 ± 3.5	1.3 ± 0.7
Late rosette	50	19 (38%)	10.3 ± 4.7	6.3 ± 3.4
Emerged	46	4 (9%)	14.0 ± 1.2	1.3 ± 0.9

Pals1 granules with and without aPKC in Control and $Par3^{Epi\Delta}$ embryos

Related to Figures S7F and S7G. Mean ± SEM per embryo, unpaired t-tests vs. Control.

Genotype (# embryos)	# Pals1 granules			# aPKC-positive Pals1 granules		
	n	per embryo	p-value	n	per embryo	p-value
Control (3)	304	101.3 ± 5.5	0.1695	37	12.3 ± 3.8	0.1634
$Par3^{Epi\Delta}$ (3)	366	122.0 ± 11.1		14	4.7 ± 2.3	
